# Supplementary material for: Prevalence of depression in China during the early stage of the COVID-19 pandemic: a cross-sectional study in an online survey sample
Source: BMJ Open. 2022 Mar 9;12(3):e056667. doi: 10.1136/bmjopen-2021-056667 (PMC8914405; doi:10.1136/bmjopen-2021-056667)
Supplement: Supplementary data [file bmjopen-2021-056667supp001.pdf]

Appendix 1

Sample size calculation with sequential methods were showed in table S1. Firstly, we make each province have 300 quotas; Next, we allocate 360 quotas to provinces that have more than 70 million; After allocating quotas according to each province's urban and sex ratio, we found that the number of the stratum Beijing-rural-male, Beijing-rural-female, Shanghai-rural-male, Shanghai-rural-female, Tianjin-rural-male, Tianjin-rural-female, Guangdong-rural-female were less than 50, so we adjusted the quotas according to the following steps:

1) We raised the quotas for Beijing-rural-male, Beijing-rural-female, Shanghai-rural-male, Shanghai-rural-female, Tianjin-rural-male, Tianjin-rural-female, Guangdong-rural-female to 50.

2) We raised the total quotas for Beijing, Tianjin and Shanghai, Zhejiang, Anhui to 360, total quotas for Liaoning to 340, thus we can achieve a goal with 10000 total sample.

3) We allocated the rest 260 quotas for Beijing-urban-male, Beijing-urban-female, Shanghai-urban-male, Shanghai-urban-female, Tianjin-urban-male, Tianjin-urban-female; and allocate the rest 310 quotas for Guangdong-urban-male, Guangdong-urban-female, Guangdong-rural-male.

Table S1 Sample size calculation with sequential methods of each province in China

City	Population (million)	Sex ratio (female=100)	City %	Rural %	Sample	City-Male	City-Female	Rural-Male	Rural-Female
Beijing	2154	98.75	86.50%	13.50%	360	133	127	50	50
Tianjing	1560	115.72	83.15%	16.85%	360	142	118	50	50
Hebei	7556	101.67	56.43%	43.57%	360	102	103	78	77
Shanxi	3718	104.73	58.41%	41.59%	300	89	87	63	61
Neimenggu	2534	104.26	62.71%	37.29%	300	95	94	57	54
Liaoning	4359	100.37	68.10%	31.90%	340	115	117	54	54
Jiling	2704	102.03	57.53%	42.47%	300	87	87	64	62
Heilongjiang	3773	103.93	60.10%	39.90%	300	91	90	61	58

Shanghai	2424	106.61	88.10%	11.90%	360	135	125	50	50
Jiangsu	8051	103.43	69.61%	30.39%	360	127	125	55	53
Zhejiang	5737	107.97	68.90%	31.10%	360	128	121	58	53
Anhui	6324	106.06	54.69%	45.31%	360	101	97	83	79
Fujian	3941	108.41	65.82%	34.18%	300	102	95	53	50
Jiangxi	4648	105.83	56.02%	43.98%	300	86	83	67	64
Shandong	10047	100.77	61.18%	38.82%	360	110	111	70	69
Henan	9605	102.45	51.71%	48.29%	360	94	94	87	85
Hubei	5917	106.15	60.30%	39.70%	300	92	90	61	57
Hunan	6899	101.51	56.02%	43.98%	300	84	85	66	65
Guangdong	11346	117.27	70.70%	29.30%	360	137	117	56	50
Guangxi	4926	108.47	50.22%	49.78%	300	78	74	77	71
Hainan	934	104.69	59.06%	40.94%	300	90	88	62	60
Chongqing	3102	100.35	65.50%	34.50%	300	98	100	51	51
Sichuan	8341	98.67	52.29%	47.71%	360	93	96	85	86
Guizhou	3600	109.37	47.52%	52.48%	300	74	69	82	75
Yunnan	4830	107.67	47.81%	52.19%	300	74	70	81	75
Xizang	344	98.98	31.14%	68.86%	300	50	50	98	102
Shanxi	3864	100.47	58.13%	41.87%	300	87	89	62	62
Gansu	2637	103.65	47.69%	52.31%	300	72	72	80	76
Qinghai	603	108.34	54.47%	45.53%	300	84	80	71	65
Ningxia	688	98.27	58.88%	41.12%	300	87	90	61	62
Xinjiang	2487	99.76	50.91%	49.09%	300	76	78	73	73
Total					10000				

Table S2 Confirmed cases of each province in China

Province	Confirmed cases
Beijing	587
Shanghai	675
Tianjing	194
Chongqing	573
Liaoning	147
Jiling	153
Heilongjiang	934
Hebei	334
Shanxi	198
Neimenggu	237
Jiangsu	653
Zhejiang	1267
Anhui	985
Fujian	357
Jiangxi	931
Shandong	785
Henan	1254
Hubei	63623
Hunan	1015
Guangdong	1627
Guangxi	252
Hainan	165
Sichuan	578
Guizhou	145
Yunnan	183
Xizang	1
Shanxi	308
Gansu	146
Qinghai	18
Ningxia	75
Xinjiang	73
