# Supplementary material for: Prevalence of depression in China during the early stage of the COVID-19 pandemic: a cross-sectional study in an online survey sample
Source: BMJ Open. 2022 Mar 9;12(3):e056667. doi: 10.1136/bmjopen-2021-056667 (PMC8914405; doi:10.1136/bmjopen-2021-056667)
Supplement: Author's manuscript [file bmjopen-2021-056667.draft_revisions.pdf]

BMJ Open

BMJ Open is committed to open peer review. As part of this commitment we make the peer review history of every article we publish publicly available.

When an article is published we post the peer reviewers' comments and the authors' responses online. We also post the versions of the paper that were used during peer review. These are the versions that the peer review comments apply to.

The versions of the paper that follow are the versions that were submitted during the peer review process. They are not the versions of record or the final published versions. They should not be cited or distributed as the published version of this manuscript.

BMJ Open is an open access journal and the full, final, typeset and author-corrected version of record of the manuscript is available on our site with no access controls, subscription charges or pay-per-view fees (http://bmjopen.bmj.com).

If you have any questions on BMJ Open's open peer review process please email info.bmjopen@bmj.com

BMJ Open

Depression in China during the coronavirus 2019 pandemic: A cross-sectional study

Journal:	*BMJ Open*
Manuscript ID	bmjopen-2021-056667
Article Type:	Original research
Date Submitted by the Author:	26-Aug-2021

Complete List of Authors:	Liu, Weina; Universitatsklinikum Heidelberg Institut fur Global Health; Jiangsu Provincial CDC,<br>Yu, Fengyun; Tsinghua University Department of Industrial Engineering; Universitatsklinikum Heidelberg Institut fur Global Health<br>Geldsetzer, Pascal; Universitatsklinikum Heidelberg Institut fur Global Health; Stanford University Department of Medicine<br>Yang, Juntao; Chinese Academy of Medical Sciences & Peking Union Medical College<br>Wang, Zhuoran; Chinese Academy of Medical Sciences & Peking Union Medical College<br>Golden, Todd; National Cancer Institute Division of Cancer Control and Population Sciences<br>Jiao, Lirui; Columbia University<br>Chen, Qiushi; The Pennsylvania State University, Harold and Inge Marcus Department of Industrial and Manufacturing Engineering<br>Liu, Haitao; Chinese Academy of Medical Sciences & Peking Union Medical College<br>Wu, Peixin; Chinese Academy of Medical Sciences & Peking Union Medical College<br>Wang, Chen; Chinese Academy of Medical Sciences & Peking Union Medical College; China-Japan Friendship Hospital<br>Bärnighausen, Till ; Universitatsklinikum Heidelberg Institut fur Global Health, Institute of Public Health; Harvard University Department of Global Health and Population<br>Chen , Simiao ; Universitatsklinikum Heidelberg Institut fur Global Health; Chinese Academy of Medical Sciences & Peking Union Medical College

Keywords:	COVID-19, Epidemiology < TROPICAL MEDICINE, MENTAL HEALTH

SCHOLARONE™
Manuscripts

I, the Submitting Author has the right to grant and does grant on behalf of all authors of the Work (as defined in the below author licence), an exclusive licence and/or a non-exclusive licence for contributions from authors who are: i) UK Crown employees; ii) where BMJ has agreed a CC-BY licence shall apply, and/or iii) in accordance with the terms applicable for US Federal Government officers or employees acting as part of their official duties; on a worldwide, perpetual, irrevocable, royalty-free basis to BMJ Publishing Group Ltd ("BMJ") its licensees and where the relevant Journal is co-owned by BMJ to the co-owners of the Journal, to publish the Work in this journal and any other BMJ products and to exploit all rights, as set out in our licence.

The Submitting Author accepts and understands that any supply made under these terms is made by BMJ to the Submitting Author unless you are acting as an employee on behalf of your employer or a postgraduate student of an affiliated institution which is paying any applicable article publishing charge ("APC") for Open Access articles. Where the Submitting Author wishes to make the Work available on an Open Access basis (and intends to pay the relevant APC), the terms of reuse of such Open Access shall be governed by a Creative Commons licence – details of these licences and which Creative Commons licence will apply to this Work are set out in our licence referred to above.

Other than as permitted in any relevant BMJ Author's Self Archiving Policies, I confirm this Work has not been accepted for publication elsewhere, is not being considered for publication elsewhere and does not duplicate material already published. I confirm all authors consent to publication of this Work and authorise the granting of this licence.

For peer review only

Depression in China during the coronavirus 2019 pandemic: A cross-sectional study

Weina Liu[1,2*], Fengyun Yu[1,3,*], Pascal Geldsetzer[1,4,*], Juntao Yang[5], Zhuoran Wang[6], Todd Golden[7], Lirui Jiao[8], Qiushi Chen[9], Haitao Liu[6], Peixin Wu[6,10], Chen Wang[6,11,12,†], Till Bärnighausen[1,6,13,#] , Simiao Chen[1,6,#,†],

[1] Heidelberg Institute of Global Health (HIGH), Faculty of Medicine and University Hospital, Heidelberg University, Heidelberg, Germany

[2] Jiangsu Province Center for Disease Control and Prevention, Nanjing, China

[3] Department of Industrial Engineering, Tsinghua University, Beijing, China

[4] Division of Primary Care and Population Health, Department of Medicine, Stanford University,-Stanford, CA, USA

[5] State Key Laboratory of Medical Molecular Biology, Institute of Basic Medical Sciences, Chinese Academy of Medical Sciences & Peking Union Medical College, Beijing, China

[6] Chinese Academy of Medical Sciences and Peking Union Medical College, Beijing, China

[7] Division of Cancer Control and Population Sciences, National Cancer Institute, Rockville, MD, USA

[8] Columbia University, New York, USA

[9] The Harold and Inge Marcus Department of Industrial and Manufacturing Engineering, The Pennsylvania State University, University Park, PA, USA

[10] Peking Union Medical College Hospital, Beijing, China

[11] National Clinical Research Center for Respiratory Diseases, Beijing, China

[12] Department of Pulmonary and Critical Care Medicine, Center of Respiratory Medicine, China–Japan Friendship Hospital, Beijing, China

[13] Department of Global Health and Population, Harvard T.H. Chan School of Public Health, Boston, MA, USA

[*] Co-first authors: WL, FY and PG contributed equally to this study

For peer review only - http://bmjopen.bmj.com/site/about/guidelines.xhtml

[#] Co-senior authors: TB and SC

[†] Co-corresponding authors:

Simiao Chen, Email: simiao.chen@uni-heidelberg.de

Chen Wang, Email: wangchen@pumc.edu.cn

For peer review only

Abstract

Objectives: We aimed to determine the prevalence of depression during the COVID-19 pandemic among Chinese adults by province, and to determine key associated sociodemographic characteristics in order to inform future focused interventions on those most affected.

Design: Cross-sectional study.

Setting: National online survey in China.

Participants: We conducted a cross-sectional online survey from May 8, 2020 to June 8, 2020, aiming for a similar distribution by sex and rural-urban residency as the general population within each of China's provinces, recruiting 300-360 adults per province (n=14,493).

Primary outcome: Participants were registered with the survey company KuRunData and completed the Patient Health Questionaire-9 (PHQ-9).We calculated the prevalence of depression (defined as a PHQ-9 score >4) nationally and separately for each province.

Secondary outcome: Covariate-unadjusted and covariate-adjusted logistic regression models were used to examine how the prevalence of depression varied by adults' sociodemographic characteristics. All analyses used survey sampling weights.

Results: The survey was initiated by 14,493 participants, with 10,000 completing all survey questions and included in the analysis. A higher risk of depression was associated with living in urban areas (OR, 1.61; 95% CI ,1.23-2.12), working as a nurse (OR, 3.54; 95% CI, 1.37-9.11), and having a family member (OR, 23.75; 95% CI, 1.86-303.51), neighbor (OR, 19.71; 95% CI, 1.54-252.22) with confirmed COVID-19. A lower risk of depression was associated with participants who had an accurate knowledge of COVID-19 transmission (OR, 0.65; 95% CI, 0.45-0.94), and awareness of recommended healthcare-seeking behavior for suspected COVID-19 (OR, 0.74; 95%CI, 0.57-0.95).The prevalence of depression in China was found to be still high (42.8%) [41.8% - 43.7%].

For peer review only - http://bmjopen.bmj.com/site/about/guidelines.xhtml

Conclusion: Depression was more common among urban dwellers, nurses, and those with a family member or neighbor with COVID-19. Accurate knowledge of COVID-19 transmission and awareness of recommended healthcare-seeking behavior may reduce the risk of depression.

Strength and limitation of this study

By sampling 10,000 individuals across China's provinces, this is the first study to assess depression of COVID-19 in each of China's provinces.

We explored how the prevalence of depression varies within provinces by individuals' socioeconomics characteristics, and thus could give indication to target groups for prevention of depression.

We systematically identified the different risk levels of depression varying by the accuracy levels of knowledge of COVID-19 and confirmed cases .

Our sample is unlikely to be representative of the general population of China, participants had to have access to a computer or smartphone and therefore trended to be younger and highly educated.

This was a cross sectional study design we can only evaluate the results from our study period itself and cannont make temporal conclusions.

For peer review only - http://bmjopen.bmj.com/site/about/guidelines.xhtml

Introduction

As of June 2021 the World Health Organziation (WHO) reports >174 million confirmed cases and >3.75 million deaths worldwide from the coronavirus disease 2019 (COVID-19) pandemic [1-3]. While vaccines are being developed and disseminated rapidly [4], with the development and spread of the epidemic, asymptomatic infection and mutated variants continue to pose an unprecedented threat. Although people in China have returned to normal life, many other countries continue to be affected by the epidemic. To reduce the impact of the pandemic, many countries imposed measures including city lockdowns, border closures, social isolation, and quarantine. Long-term lockdowns and a large number of cases may not only affect social and economic outcomes [5-10] but also disrupt normal life leading to pandemic-induced impairments in work and social functioning, including depression [11] even suicide [12].

Previous research suggests that mental health services are crucial to alleviate pandemic-related mental health impacts such as depression [12]. It is important to understand the geographic distribution of depression in order to target mental health service on a national, regional or local level. Non-pharmacuitical policies vary among countries and they change dynamically as does the prevalence of depression. Although the epidemic in China has subsided, investigating depression trends during different periods of the epidemic is imperative. A number of cross-sectional studies in different countries suggest an association between depression and sociodemographic characteristics such as female [13 14], nurses [13], low income [15], young age [14], lower education level [16], having higher COVID-19 contraction risk [17], living in urban areas, and social isolation [18]. More recently, literature has emerged that offers contradictory findings about the association between depression and these sociodemographic characteristics [17 19]. The prevalence of depression across China and the associated sociodemographic characteristics during the COVID-19 pandemic have yet to be fully understood.

For peer review only - http://bmjopen.bmj.com/site/about/guidelines.xhtml

Other factors such as whether getting infected with COVID-19, or knowledge and perceptions about the virus, may also have an impact on psychological well-being and mental health. A previous study in India showed that inadequate knowledge of COVID-19 was associated with increased mental health problems [20]. Another study provided a theoretical model of the relationship between risk perception of COVID-19, coping and psychological well-being and found the perception of COVID-19 may predict psychological well-being through adoption of different types of coping strategies such as problem-focused coping and emotion-focused coping [21]. Increased knowledge regarding COVID-19 may contribute to better coping strategies, which in turn, affect mental health [22].

This study, for the first time, addresses how the prevalence of depression varies by regions, sociodemographic characteristics, and other important factors that could affect psychological well-being and mental health, such as previous infections and knowledge and perceptions about the virus. These findings may help formulate effective interventions for improving mental health during different periods of the COVID-19 pandemic in different regions.

For peer review only - http://bmjopen.bmj.com/site/about/guidelines.xhtml

Methods

Study Design and Sampling

We conducted a cross-sectional online survey from May 8, 2020 to June 8, 2020. The survey was carried out with Kurun Information Technology Co. Ltd. (KuRunData, Shanghai, China). We sampled 10,000 adults in total (300-360 participants from 31 provincial administrative units in China). Within each province, we aimed to reflect the province's population composition by gender and urban and rural residence (according to the China Statistical Yearbook 2020 [23]). Participants were paid 5 yen (US$0.77) for completing the questionnaire. All pariticpants completed informed written consent.

Patient and Public Involvement

None of the participants was involved in the design of the questionnaire, nor the design, recruitment, and implementation of the study. We plan to disseminate our study widely through the media, including in China.

Measurements and Covariates

The questionnaire included three parts: 1) COVID-19 awareness (perceived risk of death from COVID-19, knowledge of the transmission of COVID-19, awareness of recommended healthcare-seeking behavior; 2) sociodemographic information (sex, age, highest education level, ethnicity, residence type, health care providers, annual household income, personal COVID-19 dignosis history, positive acquaintance COVID-19 diagnosis; and 3) the Patient Health Questionnaire-9 (PHQ-9; a depression screening tool [24]). We defined a score $\geq$4 as depression. COVID-19 awareness consisted of 9 items (perceived risk of death among vulnerable groups, perceived risk of death among people with other diseases, perceived elderly as a high-risk group of transmission, awareness of vaccine availability protecting against transmission, awareness that masks are highly effective in protecting against transmission, knowledge of transmission prevention actions, knowledge of saliva as main

For peer review only - http://bmjopen.bmj.com/site/about/guidelines.xhtml

transmission route,awareness of COVID-19 symptoms,and awareness of recommended healthcare-seeking behavior). Among these nine items, for categorical outcomes , data are expressed as binary or categorical (range: 0 -100%). For continuous outcomes, data are expressed as median (interquartile range). Our data on knowledge of COVID-19 was previously published [25].

Statistical Analysis

We excluded questionnaire which participants answered less than half of the questions. All analyses applied sampling weights after excluding incomplete questionnaires. The sampling weights were calculated by the inverse of the probability of age, residence type, and sex of selecting participants. These probabilities were obtained using sampling weights from population counts from the 2019 census in China. For binary and categorical response options of knowledge about COVID-19, we calculated the percentage of participants with correct responses. For binomial proportions, we produced 95% confidence intervals by the Wilson score interval. The prevalence of depression was calculated by the participants' sociodemographic characteristics and overall knowledge about COVID-19 using χ^2 to compare. To examine how depression prevalence varied by sociodemographic characteristics, we used covariate-unadjusted and covariate-adjusted logistic regression with a binary indicator for each province (province-level fixed effects) and obtained an odds ratio (OR). All statistical analyses were conducted in R version 4.0.3.

For peer review only - http://bmjopen.bmj.com/site/about/guidelines.xhtml

Results

Sociodemographic characteristics

The online survey was initiated by 14,493 participants, with 10,000 completing all survey questions and taking more than two minutes, taking less than this time cutoff was considered insuffcent to read and answer all questions.

50.8% of participants were male. Except for 9% of participants aged under 20 years, the percentage of other age groups ranged from 16.5% to 19.0%. Participants with high school education level and above accounted for 73.1% of the sample. Han ethnicity accounted for the vast majority of participants (93.8%). 59.4% of participants lived in urban areas. Health care providers account for 4% of participants, with the largest category being community health workers (1.6%). 53.7% of participants had an annual household income ≥90,000 RMB (~US $13,000). 0.08% of participants had been diagnosed with COVID-19 and 0.34% of participants knew someone diagnosed with COVID-19. 42.8% of participants reported to have been diagnosed with depression. The correct rate of COVID-19 awareness ranged from 11.1% to 96.3%, knowledge of COVID-19 transmission prevention actions was lowest (11.1%[95% CI: 10.5% - 11.7%]) and knowledge about perceived risk of COVID-19 death among people with other diseases was highest (96.3%[95% CI: 96.0% - 96.7%])(Table 1).

Variation of depression prevalence by province

As seen in Figure 1, the prevalence of depression (PHQ-9 score >4) in southern China tended to be greater than in northern China. The overall prevalence of depression was 42.8% (95% CI: 41.8%-43.7%). The prevalence of COVID-19 in Hubei province (1,148 per million) tended to be higher than other areas in China during the same time period, and Tibet (0.28 per million) was the lowest province. While the prevalence of COVID-19 cases from other provinces tended to be the same except for Hubei and Tibet (Figure 2).

For peer review only - http://bmjopen.bmj.com/site/about/guidelines.xhtml

Variation of depression prevalence by sociodemographic characteristics within provinces

The results of covariate-unadjusted and covariate-adjusted logistic regression analysis of associated factors with depression are shown in Table2. After adjusting for covariates, participants living in urban areas had higher odds (OR, 1.61; 95% CI, 1.23-2.12) of depression than those in rural areas. In addition, nurses had higher odds of depression than other health care providers (OR, 3.54; 95%CI, 1.37-9.11), and having a family member(OR, 23.75; 95% CI, 1.86-303.51) or neighbor (OR, 19.71; 95% CI, 1.54 - 252.22) with confirmed COVID-19 increased the odds of depression significantly . Participants who had correct knowledge of COVID-19 transmission prevention actions had lower odds of depression (OR, 0.65; 95% CI, 0.45-0.94), as did participants who had accurate awareness of recommended healthcare-seeking behavior (OR, 0.74; 95% CI, 0.57-0.95).

For peer review only

Table 1. Sociodemographic Characteristics, COVID-19 Awareness, and Depression Score (PHQ-9) (10,000 survey participants)

Characteristic	Proportion (Weighted)[1]	N (%) (Not weighted)	Proportion[2]
Sex			
Female	48.9%	4,921 (49.2%)	48.9%
Age Group			
< 20 Years	10.5%	900 (9.0%)	6.9%
20-29 Years	17.1%	1,645 (16.5%)	20.8%
30-39 Years	17.9%	1,895 (19.0%)	18.2%
40-49 Years	18.9%	1,890 (18.9%)	22.1%
50-59 Years	18.0%	1,820 (18.2%)	16.5%
≥ 60 Years	17.5%	1,675 (16.8%)	15.4%
Highest Education Level			
None	4.0%	438 (4.4%)	5.4%
Elementary School	4.2%	475 (4.8%)	25.3%
Middle School	16.6%	1,779 (17.8%)	37.8%
High School / Technical Secondary School	37.1%	3,733 (37.3%)	17.6%
College / Undergraduate	36.0%	3,369 (33.7%)	13.4%
Graduate and Above	2.1%	206 (2.0%)	0.6%
Ethnicity			
Han	95.1%	9,381 (93.8%)	95.0%
Man	0.5%	149 (1.5%)	0.7%
Hui	0.1%	109 (1.1%)	0.8%
Zang	1.8%	103 (1.0%)	0.5%
Zhuang	1.4%	152 (1.5%)	1.2%
Other	1.1%	106 (1.1%)	1.8%
Province of Residence			
Anhui	4.5%	360 (3.6%)	4.5%
Beijing	1.5%	360 (3.6%)	1.5%
Chongqing	2.2%	360 (3.6%)	2.2%
Fujian	2.8%	300 (3.0%)	2.8%
Gansu	1.9%	300 (3.0%)	1.9%
Guangdong	8.2%	360 (3.6%)	8.2%
Guangxi	3.5%	300 (3.0%)	3.5%
Guizhou	2.6%	300 (3.0%)	2.6%
Hainan	0.7%	300 (3.0%)	0.7%
Hebei	5.4%	360 (3.6%)	5.4%
Heilongjiang	2.7%	300 (3.0%)	2.7%
Henan	6.9%	360 (3.6%)	6.9%
Hubei	4.2%	360 (3.6%)	4.2%
Hunan	4.9%	300 (3.0%)	4.9%
Jiangsu	5.7%	360 (3.6%)	5.7%
Jiangxi	3.3%	300 (3.0%)	3.3%
Jilin	1.9%	300 (3.0%)	1.9%
Liaoning	3.1%	340 (3.4%)	3.1%
Neimengol	1.8%	300 (3.0%)	1.8%
Ningxia	0.5%	300 (3.0%)	0.5%
Qinghai	0.4%	300 (3.0%)	0.4%
Shaanxi	2.7%	360 (3.6%)	2.7%
Shandong	7.2%	360 (3.6%)	7.2%
Shanghai	1.7%	300 (3.0%)	1.7%
Shanxi	2.8%	300 (3.0%)	2.8%
Sichuan	6.0%	360 (3.6%)	6.0%
Tianjin	1.1%	360 (3.6%)	1.1%
Tibet	0.3%	300 (3.0%)	0.3%
Xinjiang	1.8%	300 (3.0%)	1.8%

For peer review only - http://bmjopen.bmj.com/site/about/guidelines.xhtml

Yunnan	3.5%	300 (3.0%)	3.5%
Zhejiang	4.2%	360 (3.6%)	4.2%
Residence Type			
Urban	61.1%	5,935 (59.4%)	60.6%
Health Care Providers			
No	96.0%	9,597 (96.0%)	99.0%
Nurse	0.5%	55 (0.6%)	0.3%
Physician	0.9%	84 (0.8%)	0.5%
Community Health Worker	1.5%	157 (1.6%)	<0.1%
Pharmacist	0.1%	17 (0.2)	<0.1%
Other Healthcare Provider	1.0%	90 (0.9%)	0.1%
Annual Household Income (RMB)			
< 30,000	5.8%	560 (5.6%)	
30,000 - 59,999	14.9%	1,670 (16.7%)	-
60,000 - 89,999	21.6%	2,303 (23.0%)	-
90,000 - 119,999	26.2%	2,704 (24.0%)	-
120,000 - 149,999	14.2%	1,211 (12.1%)	-
150,000 - 199,999	11.1%	974 (9.7%)	-
≥ 200,000	6.4%	578(5.8%)	-
Personal COVID-19 Diagnosis History			
No	99.9%	9,992 (99.9%)	
Yes	0.11%	8(0.08%)	
Postive Acquaintance COVID-19 Diagnosis			
Family member	0.07%	5(0.05%)	
Friend	0.14%	12(0.12%)	
Neighbor	0.09%	7(0.07%)	
Cowoker	0.13%	10(0.1%)	
Other	0%	1(0)	
Depression By Screening Tool (PHQ-9)			
No (PHQ-9 ≤ 4)	56.1%	5,723(57.2%)	
Yes (PHQ-9 ≥4)	43.9%	4,277(42.8%)	
COVID-19 Awareness			
Perceived risk of death among vulnerable groups	3.3% (IQR: 1.0%-4.0%)	3.3% (IQR: 1.0%-4.0%)	
Perceived risk of death among people with other diseases	96.4% (95% CI: 96.0% - 96.8%)	96.3% (95% CI: 96.0% - 96.7%)	
Perceived *elderly* as *a high-risk group of transmission*	92.6% (95% CI: 92.0% - 93.1%)	92.7% (95% CI: 92.2% - 93.2%)	
Awareness of *vaccine* availability *protecting against transmission*	76.5% (95% CI: 75.7% - 77.3%)	76.0% (95% CI: 75.1% - 76.8%)	
Awareness that *masks* are *highly effective in protecting against transmission*	89.2% (95% CI: 88.6% - 89.8%)	89.8% (95% CI: 89.2% - 90.4%)	
Knowledge of *transmission* prevention actions	11.4% (95% CI: 10.8% - 12.0%)	11.1% (95% CI: 10.5% - 11.7%)	
Knowledge of *saliva* as main transmission route	82.2% (95% CI: 81.5% - 83.0%)	82.1% (95% CI: 81.3% - 82.8%)	
Awareness *of COVID-19* symptoms	69.1% (95% CI: 68.2% - 70.0%)	67.1% (95% CI: 66.1% - 68.0%)	
Awareness of *recommended healthcare-seeking behavior*	71.4% (95% CI: 70.5% - 72.3%)	68.8% (95% CI: 67.9% - 69.7%)	

[1] Weighted using survey sampling weights.

[2] As per the 2020 China Statistical Yearbook.

For dichotomous outcomes, data are expressed as a percentage with the correct response (95% confidence interval). For continuous outcomes, data are expressed as median (interquartile range)

For peer review only - http://bmjopen.bmj.com/site/about/guidelines.xhtml

Table 2. Covariate-unadjusted and covariate-adjusted logistic regressions of depression onto sociodemographic characteristics

	Covariate-unadjusted OR (95% CI) [1]	Covariate-adjusted OR (95% CI)[2]
Overall		
Sex		
Male	Ref.	Ref.
Female	0.87 (0.72 - 1.07)	0.88 (0.72 – 1.08)
Age		
18-19 years	Ref.	Ref.
20-29 years	1.03 (0.71 - 1.52)	1.09 (0.71 – 1.67)
30-39 years	0.81 (0.54 - 1.2)	0.86 (0.54 – 1.36)
40-49 years	1.07 (0.73 - 1.56)	1.09 (0.72 – 1.66)
50-59 years	1.07 (0.73 - 1.57)	0.99 (0.65 – 1.52)
>60 years	0.83 (0.56 - 1.24)	0.81 (0.48 – 1.35)
Highest Education Level		
None	Ref.	Ref.
Elementary school	1.39 (0.66 - 2.91)	1.30 (0.62 - 2.74)
Middle school	1.72 (0.93 - 3.16)	1.43 (0.74 - 2.76)
High school / Technical secondary school	1.51 (0.83 - 2.73)	1.19 (0.59 - 2.38)
College / Undergraduate	1.44 (0.80 - 2.62)	1.12 (0.56 - 2.26)
Graduate and Above	1.51 (0.61 - 3.77)	1.23 (0.45 - 3.32)
Residence Type		
Rural	Ref.	Ref.
Urban	1.11 (0.90 - 1.37)	1.61 (1.23 - 2.12)***
Health Care Providers		
No	Ref.	Ref.
Nurse	2.96 (1.15 - 7.64)*	3.54 (1.37 - 9.11)**
Physician	1.53 (0.59 - 3.94)	1.53 (0.6 - 3.9)
Community health worker	1.6 (0.84 - 3.03)	1.52 (0.8 - 2.9)
Pharmacist	0.91 (0.16 - 5.1)	1.21 (0.22 - 6.55)
Other healthcare provider	1.22 (0.52 - 2.85)	1.12 (0.46 - 2.76)
Annual household income (RMB)		
< 30,000	Ref.	Ref.
30,000 – 59,999	0.92 (0.58 - 1.47)	0.98 (0.6 - 1.59)
60,000 – 89,999	0.76 (0.48 - 1.2)	0.8 (0.49 - 1.3)
90,000 – 119,999	1.09 (0.7 - 1.7)	1.18 (0.72 - 1.91)
120,000 – 149,999	0.85 (0.52 - 1.38)	0.89 (0.52 - 1.53)
150,000 – 199,999	0.67 (0.39 - 1.13)	0.68 (0.38 - 1.23)
≥ 200,000	0.68 (0.37 - 1.24)	0.7 (0.36 - 1.36)
COVID-19 Awareness		

Perceived risk of death among vulnerable groups	1.02 (0.99 - 1.06)	1.03 (1 - 1.06)
Perceived risk of death among people with other diseases	0.89 (0.72 - 1.1)	0.9 (0.73 - 1.12)
Perceived elderly as a high-risk group of transmission	1.12 (0.76 - 1.64)	1.17 (0.79 - 1.72)
Awareness of vaccine availability protecting against transmission	0.88 (0.7 - 1.11)	0.86 (0.67 - 1.1)
Awareness that masks are highly effective in protecting against transmission	0.96 (0.7 - 1.31)	0.98 (0.72 - 1.34)
Knowledge of transmission prevention actions	0.63 (0.44 - 0.91)*	0.65 (0.45 - 0.94)*
Knowledge of saliva as main transmission route	0.75 (0.59 - 0.96)*	0.82 (0.64 - 1.05)
Awareness of COVID-19 symptoms	0.81 (0.65 - 1.00)	0.83 (0.67 - 1.04)
Awareness of recommended healthcare -seeking behavior	0.78 (0.63 - 0.98)*	0.74 (0.57 - 0.95)*
Postive Acquaintance COVID-19 Diagnosis		
No	Ref.	Ref.
Self	0.89 (0.03 - 28.47)	0.51 (0.03 - 9.46)
Family member	17.73 (1.36 - 230.99)	23.75 (1.86 - 303.51)*
Neighbor	19.61 (1.58 - 243.79)	19.71 (1.54 - 252.22)*
Coworker	4.2 (0.59 - 29.64)	4.62 (0.62 - 34.23)
Friend	0.59 (0.11 - 3.12)	0.62 (0.12 - 3.22)
COVID-19 cases confirmed by the province		
Prevalence of COVID-19 by province	0.97 (0.85 - 1.11)	0.98 (0.85 - 1.13)

1. Covariate-unadjusted logistic Regression results [All regressions included each one of the variables shown in the table and adjusted for each province (province-level fixed effects)]

2. Covariate-adjusted logistic Regression results [All regressions included all variables shown in the table and adjusted for each province (province-level fixed effects)]

3. ***represents *p<0.001;* **represents *p<0.01;* * represents *p<0.05*

Discussion

This is the first study that quantified the prevalence of depression symptoms and its variation by province and sociodemographic characteristics in China. Around 42.8% of participants in our sample had depression as defined by a PHQ-9 score greater than 4. Our research not only described the prevalence of depression in China but also found a higher prevalence of depression among certain population groups, including urban dwellers, nurses, and those with a family member or neighbor with confirmed COVID-19. Furthermore, we found that knowledge and perceptions of COVID-19 may have a "protective effect" on mental health, indicating the urgency and importance of effective communication of education amid the

For peer review only - http://bmjopen.bmj.com/site/about/guidelines.xhtml

COVID-19 pandemic. These findings may aid in the development of intervention strategies for different populations.

Several studies have described the prevalence of depression during the COVID-19 pandemic in the general population. The prevalence of depression observed in our study was lower than a national study among 4,872 participants conducted January 31 to February 2, 2020 in China (48.3%) [26], but higher than that in a previous study of 71,227 individuals among general population during the COVID-19 outbreak in China from February 28 to March 11, 2020 (27.9%) [27] and prior to the COVID-19 epidemic (4.3%) [28]. Most of these other studies were conducted in February 2020─at the peak of the COVID-19 epidemic [26 29-34], which ranged from 8.3 to 48.3% [26 29-34]. Although our study occured in May when the epidemic had decreased in severity, the prevalence of depression had not been alleviated, which – in addition to differences in sampling strategy – may explain why the prevalence of depression observed was still high. Another possible reason may be that under continuous strict quarantine policy, exposure to COVID-19 related news and social media was associated with depression [35 36].

The prevalence of COVID-19 from 31 provinces tended to be similar except for Hubei which had the highest prevalence and Tibet which had the lowest one (Figure 2). However, the proportion of the population by province suffering from depression (PHQ-9>4) did not seem to be significantly associated with the prevalence of COVID-19 cases. Moreover, from the covariate-adjusted logistic regression results, we found that depression was not associated with the prevalence of COVID-19 cases confirmed by province, which also suggest that the prevalence of depression is not associated with the severity of the regional epidemic during the COVID-19 outbreak. One explanation is that resilience plays a protective role in mitigating the impact of stress and trauma on depressive symptoms and the consequences associated with

For peer review only - http://bmjopen.bmj.com/site/about/guidelines.xhtml

depressive symptoms [37]. During the initial phase of the COVID-19 outbreak in China, the Chinese government enforced a rigid social distancing policy and strongly promoted hand washing, surface disinfection, and the use of protective masks through social media [38]. In Wuhan, as the worst-hit area, people with mild and asymptomatic infection were taken care of in Fangcang shelter hospitals, for facility-based isolation, treatment, and monitoring [38]. Facility-based isolation is effective in curbing the epidemic [39 40]. Fangcang shelter hospitals also provided mental health counselling services and social support to help patients recover during isolation [41], which may also reduce the incidence of depression in hard-hit areas. Due to very limited confirmed cases in our study it was difficult to investigate the association between depression and the number of confirmed cases (0.13% participants diagnosed with COVID-19, 0.48% participants with an acquaintance with confimed COVID-19).

This study, for the first time, provides preliminary evidence that knowledge of COVID-19 transmission prevention actions and awareness of recommended healthcare-seeking behavior may be associated with a lower risk of depression. Compared to previous research [2 34 42], our study reinforced the positive association between precautionary measures and depression and explored the correlation between awareness of recommended healthcare-seeking behavior and depression. One possible reason could be explained that accurate knowledge of COVID-19 transmission prevention actions and awareness of recommended healthcare-seeking behavior may help people alleviate the stress caused by fear of COVID-19 pandemic. Previous study about menstrual hygiene management among adolescent schoolgirls indicates that inadequate knowledge could easily lead to poor behavior which can result in psychological stress [43 44]. Stress, as a risk factor for depression, plays a role in triggering depression by biological mechanisms such as HPA axis stress response processes and hormonal and neurotransmitter systems [45]. Therefore, accurate knowledge and perceptions towards COVID-19 could

For peer review only - http://bmjopen.bmj.com/site/about/guidelines.xhtml

indirectly affect depression through reduction in stress. This finding suggests that, to reduce the prevalence of depression, a effective communiation and education of COVID-19 preventive measures and recommended healthcare-seeking behaviors is urgently needed.

The correlation between urban residence and depression was positive in our study, which is consistent with several previous studies [46 47]. A possible interpretation of this finding is that while the virus could be transmitted more quickly in urban areas with a higher density population [48], those in urban areas tend to have higher education levels as well as greater access to COVID-19 information [46]. Moreover, the social distancing restrictions allowed less travel in cities than in rural areas, potentially contributing to the higher prevalence of depression in urban than in rural areas. Our study also found that nurses were more susceptible to depression, consistent with previous studies [31 49-51]. Moreover, we found those with a family member or neighbor with confirmed COVID-19 more vulnerable to depression, consistent with previous findings suggesting having a high risk of contracting COVID-19 contributes to the likelihood of depression [52 53]. These findings reinforce previous studies and suggest the importance of establishing effective intervention strategies focusing on vulnerable populations.

Limitations

This is the first study that investigated the prevalence of depression by province during the initial stage of the COVID-19 pandemic in China; however, our study has several limitations. First, although our sample size was large all participants needed to register with KuRunData therefore our sample is unlikely to be representative of the general population of China. As this was an online survey, participants had to have access to a computer or smartphone and therefore trended to be younger and highly educated. Also, our measure of depression was the PHQ-9 score, which is a tool for screening for depression but is not recommended for a clinical

For peer review only

diagnosis of the condition. Additionally, as this was a cross sectional study design we can only evaluate the results from our study period itself and cannont make temporal conclusions.

Conclusions

In conclusion, the prevalence of depression in China during the initial stage of the COVID-19 pandemic was high. Depression was more common among urban dwellers, nurses, and those with a family member or neighbor confirmed with COVID-19. Accurate knowledge of COVID-19 transmission and awareness of the recommended healthcare-seeking behavior was associated with a lower risk of depression.These findings may assist with targeted efforts to prevent or treat depression in China to those most in need of mental health services during the COVID-19 pandemic.

Contributors

WL and SC conceived the idea, JY, ZW, HL,PW, collected data, FY and WL analyzed and verified data, WL wrote the first draft, PG and TG edited the manuscript, LJ, QC, SC, CW and TB collaboratively reviewed the manuscript.

Funding

This work was supported by the Bill & Melinda Gates Foundation (Project INV-006261) and the Sino- German Center for Research Promotion (Project C-0048). The funding source had involvement in study design and data collection.

Competing interests

The authors have declared no conflicts of interest.

Patient consent for publication

Not required.

Ethics Approval

This research was approved by the institutional review board of Heidelberg University Hospital, and authors had access only to de-identified data. According to the rules of the institutional

For peer review only - http://bmjopen.bmj.com/site/about/guidelines.xhtml

review board of the University Hospital Heidelberg, this study was exempt for not-human-subjects research because KuRunData only shared pseudonymized data with the researchers.

Data sharing statement

The primary dataset that reported in this publication will be available but need to obtain permission from the investigative institution. Extra data is available by emailing the corresponding author Simiao Chen.

References

1. World Health Organization. Coronavirus disease (COVID-19) pandemic [Available from: https://covid19.who.int/?gclid=Cj0KCQjw8IaGBhCHARIsAGIRRYpDW4NS0CUiiW6a4fYWJ-AdgKZk4MDgbs9ck5gmi1c21JXMESfvjZwaAkP4EALw_wcB accessed 10 June 2021.
2. Wang C, Pan R, Wan X, et al. A longitudinal study on the mental health of general population during the COVID-19 epidemic in China. *Brain, behavior, immunity* 2020;87:40-48.
3. World Health Organization. Coronavirus disease (COVID-19) pandemic [Available from: https://www.who.int/emergencies/diseases/novel-coronavirus-2019?gclid=Cj0KCQiAst2BBhDJARIsAGo2ldULU-2EnH-n_1ybuEv2jPrR3bONMUWXeZqzbPtFyBco5lF99c_Xa1UaAu5uEALw_wcB accessed 25 February 2021.
4. Hu Y, Chen S. What can we learn from COVID-19 vaccine R&D in China? A discussion from a public policy perspective. *Journal of Travel Medicine* 2021;28(4) doi: 10.1093/jtm/taab026
5. Wang C, Horby PW, Hayden FG, et al. A novel coronavirus outbreak of global health concern. *The lancet* 2020;395(10223):470-73.
6. Roberton T, Carter ED, Chou VB, et al. Early estimates of the indirect effects of the COVID-19 pandemic on maternal and child mortality in low-income and middle-income countries: a modelling study. *The Lancet Global Health* 2020;8(7):e901-e08.
7. Cutler DM, Summers LH. The COVID-19 pandemic and the $16 trillion virus. *Jama* 2020;324(15):1495-96.
8. Do D, Sarker M, Chen S, et al. Healthcare worker attendance during the early stages of the COVID-19 pandemic: A longitudinal analysis of fingerprint-verified data from all public-sector secondary and tertiary care facilities in Bangladesh. *Journal of Global Health* 2020;10(2)
9. Chen S, Prettner K, Kuhn M, et al. The economic burden of COVID-19 in the United States: Estimates and projections under an infection-based herd immunity approach. *The Journal of the Economics of Ageing* 2021:100328. doi: https://doi.org/10.1016/j.jeoa.2021.100328
10. Prettner K, Chen S, Kuhn M, et al. Effective pandemic management that minimises economic harm. *Europe* 2021
11. Bao Y, Sun Y, Meng S, et al. 2019-nCoV epidemic: address mental health care to empower society. *The Lancet* 2020;395(10224):e37-e38.
12. Dawel A, Shou Y, Smithson M, et al. The Effect of COVID-19 on Mental Health and Wellbeing in a Representative Sample of Australian Adults. *Frontiers in psychiatry*

For peer review only - http://bmjopen.bmj.com/site/about/guidelines.xhtml

2020;11:579985. doi: 10.3389/fpsyt.2020.579985 [published Online First: 2020/11/03]
13. Batra K, Singh TP, Sharma M, et al. Investigating the Psychological Impact of COVID-19 among Healthcare Workers: A Meta-Analysis. *International journal of environmental research and public health* 2020;17(23) doi: 10.3390/ijerph17239096 [published Online First: 2020/12/10]
14. Al Zabadi H, Alhroub T, Yaseen N, et al. Assessment of Depression Severity During Coronavirus Disease 2019 Pandemic Among the Palestinian Population: A Growing Concern and an Immediate Consideration. *Frontiers in psychiatry* 2020;11:570065. doi: 10.3389/fpsyt.2020.570065 [published Online First: 2021/01/12]
15. Sazakli E, Leotsinidis M, Bakola M, et al. Prevalence and associated factors of anxiety and depression in students at a Greek university during COVID-19 lockdown. *Journal of public health research* 2021 doi: 10.4081/jphr.2021.2089 [published Online First: 2021/05/08]
16. Lei L, Huang X, Zhang S, et al. Comparison of prevalence and associated factors of anxiety and depression among people affected by versus people unaffected by quarantine during the COVID-19 epidemic in Southwestern China. *J Medical science monitor: international medical journal of experimental clinical research* 2020;26:e924609-1.
17. Zhang W-r, Wang K, Yin L, et al. Mental health and psychosocial problems of medical health workers during the COVID-19 epidemic in China. *Psychotherapy psychosomatics* 2020;89(4):242-50.
18. Durankuş F, Aksu E. Effects of the COVID-19 pandemic on anxiety and depressive symptoms in pregnant women: a preliminary study. *The journal of maternal-fetal & neonatal medicine : the official journal of the European Association of Perinatal Medicine, the Federation of Asia and Oceania Perinatal Societies, the International Society of Perinatal Obstet* 2020:1-7. doi: 10.1080/14767058.2020.1763946 [published Online First: 2020/05/19]
19. Zhang WR, Wang K, Yin L, et al. Mental Health and Psychosocial Problems of Medical Health Workers during the COVID-19 Epidemic in China. *Psychotherapy and psychosomatics* 2020;89(4):242-50. doi: 10.1159/000507639 [published Online First: 2020/04/10]
20. Mishra P, Bhadauria US, Dasar PL, et al. Knowledge, attitude and anxiety towards pandemic flu a potential bio weapon among health professionals in Indore City. *Przeglad epidemiologiczny* 2016;70(1):41-5, 125.
21. Krok D, Zarzycka B. Risk Perception of COVID-19, Meaning-Based Resources and Psychological Well-Being amongst Healthcare Personnel: The Mediating Role of Coping. *Journal of clinical medicine* 2020;9(10):3225.
22. Floyd DL, Prentice-Dunn S, Rogers RW. A meta-analysis of research on protection motivation theory. *Journal of applied social psychology* 2000;30(2):407-29.
23. National Bureau of Statistics of China. The urban-rural composition of the population by region and the birth rate, death rate, and natural growth rate（2019） Beijing2020 [cited 2021 May 30]. Available from: http://www.stats.gov.cn/tjsj/ndsj/2020/indexch.htm accessed May 30 2021.
24. Kroenke K, Spitzer RL. The PHQ-9: A New Depression Diagnostic and Severity Measure. *Psychiatr Ann* 2002;32(9):509-21.
25. Yu F, Geldsetzer P, Meierkord A, et al. Knowledge About COVID-19 Among Adults in China: Cross-sectional Online Survey. *Journal of Medical Internet Research* 2021;23(4):e26940.

For peer review only - http://bmjopen.bmj.com/site/about/guidelines.xhtml

26. Gao J, Zheng P, Jia Y, et al. Mental health problems and social media exposure during COVID-19 outbreak. *Plos one* 2020;15(4):e0231924.
27. Shi L, Lu ZA, Que JY, et al. Prevalence of and Risk Factors Associated With Mental Health Symptoms Among the General Population in China During the Coronavirus Disease 2019 Pandemic. *JAMA Netw Open* 2020;3(7):e2014053. doi: 10.1001/jamanetworkopen.2020.14053 [published Online First: 2020/07/02]
28. Qin X, Wang S, Hsieh C-R. The prevalence of depression and depressive symptoms among adults in China: estimation based on a National Household Survey. *China Economic Review* 2018;51:271-82.
29. Huang Y, Zhao N. Generalized anxiety disorder, depressive symptoms and sleep quality during COVID-19 outbreak in China: a web-based cross-sectional survey. *Psychiatry research* 2020;288:112954.
30. Wang C, Pan R, Wan X, et al. A longitudinal study on the mental health of general population during the COVID-19 epidemic in China. *Brain, behavior,* 2020;87:40-48.
31. Qiu J, Shen B, Zhao M, et al. A nationwide survey of psychological distress among Chinese people in the COVID-19 epidemic: implications and policy recommendations. *General psychiatry* 2020;33(2)
32. Ahmed MZ, Ahmed O, Aibao Z, et al. Epidemic of COVID-19 in China and associated psychological problems. *Asian journal of psychiatry* 2020;51:102092.
33. Zhou S-J, Zhang L-G, Wang L-L, et al. Prevalence and socio-demographic correlates of psychological health problems in Chinese adolescents during the outbreak of COVID-19. *European Child Adolescent Psychiatry* 2020;29(6):749-58.
34. Wang C, Pan R, Wan X, et al. Immediate psychological responses and associated factors during the initial stage of the 2019 coronavirus disease (COVID-19) epidemic among the general population in China. *International journal of environmental research public health* 2020;17(5):1729.
35. Huang Y, Zhao N. Generalized anxiety disorder, depressive symptoms and sleep quality during COVID-19 outbreak in China: a web-based cross-sectional survey. *Psychiatry Res* 2020;288:112954. doi: 10.1016/j.psychres.2020.112954 [published Online First: 2020/04/24]
36. Ni MY, Yang L, Leung CMC, et al. Mental Health, Risk Factors, and Social Media Use During the COVID-19 Epidemic and Cordon Sanitaire Among the Community and Health Professionals in Wuhan, China: Cross-Sectional Survey. *JMIR Ment Health* 2020;7(5):e19009. doi: 10.2196/19009 [published Online First: 2020/05/05]
37. Bitsika V, Sharpley CF, Bell R. The buffering effect of resilience upon stress, anxiety and depression in parents of a child with an autism spectrum disorder. *Journal of Developmental Physical Disabilities* 2013;25(5):533-43.
38. Chen S, Yang J, Yang W, et al. COVID-19 control in China during mass population movements at New Year. *The Lancet* 2020;395(10226):764-66.
39. Chen S, Chen Q, Yang J, et al. Curbing the COVID-19 pandemic with facility-based isolation of mild cases: a mathematical modeling study. *Journal of Travel Medicine* 2020;28(2) doi: 10.1093/jtm/taaa226
40. Dickens BL, Koo JR, Wilder-Smith A, et al. Institutional, not home-based, isolation could contain the COVID-19 outbreak. *The Lancet* 2020;395(10236):1541-42.
41. Chen S, Zhang Z, Yang J, et al. Fangcang shelter hospitals: a novel concept for responding to public health emergencies. *The Lancet* 2020;395(10232):1305-14.
42. Qian Y, Wu K, Xu H, et al. A survey on physical and mental distress among cancer patients during the COVID-19 epidemic in Wuhan, China. *Journal of palliative medicine* 2020;23(7):888-89.

For peer review only - http://bmjopen.bmj.com/site/about/guidelines.xhtml

43. Phillips-Howard PA, Caruso B, Torondel B, et al. Menstrual hygiene management among adolescent schoolgirls in low-and middle-income countries: research priorities. *Global health action* 2016;9(1):33032.
44. Sumpter C, Torondel B. A systematic review of the health and social effects of menstrual hygiene management. *PloS one* 2013;8(4):e62004.
45. Hammen CL. Stress and depression: old questions, new approaches. *Current Opinion in Psychology* 2015;4:80-85.
46. Özdin S, Bayrak Özdin Ş. Levels and predictors of anxiety, depression and health anxiety during COVID-19 pandemic in Turkish society: The importance of gender. *International Journal of Social Psychiatry* 2020;66(5):504-11.
47. Chen Y, Jin Y, Zhu L, et al. The network investigation on knowledge, attitude and practice about COVID-19 of the residents in Anhui Province. *Zhonghua yu fang yi xue za zhi* 2020;54(4):367-73.
48. Taylor S. The psychology of pandemics: Preparing for the next global outbreak of infectious disease: Cambridge Scholars Publishing 2019.
49. Chou L-P, Li C-Y, Hu SC. Job stress and burnout in hospital employees: comparisons of different medical professions in a regional hospital in Taiwan. *BMJ open* 2014;4(2)
50. Sareen J, Erickson J, Medved MI, et al. Risk factors for post-injury mental health problems. *Depression anxiety* 2013;30(4):321-27.
51. Luo M, Guo L, Yu M, et al. The psychological and mental impact of coronavirus disease 2019 (COVID-19) on medical staff and general public–A systematic review and meta-analysis. *Psychiatry research* 2020:113190.
52. Ni MY, Yang L, Leung CM, et al. Mental health, risk factors, and social media use during the COVID-19 epidemic and cordon sanitaire among the community and health professionals in Wuhan, China: cross-sectional survey. *JMIR mental health* 2020;7(5):e19009.
53. Mazza C, Ricci E, Biondi S, et al. A nationwide survey of psychological distress among Italian people during the COVID-19 pandemic: immediate psychological responses and associated factors. *International journal of environmental research public health* 2020;17(9):3165.

For peer review only

Figure 1. Proportion of the population reporting depression (PHQ-9 >4) by province[*]

(10,000 participants, May 8-June 8, 2020)

[*] Taiwan, Hong Kong and Macao are shown in grey.

Figure 2 . Prevalence of COVID-19 by province[*] (January 1-June 8, 2020)

[*] Taiwan, Hong Kong and Macao are shown in grey.

For peer review only - http://bmjopen.bmj.com/site/about/guidelines.xhtml

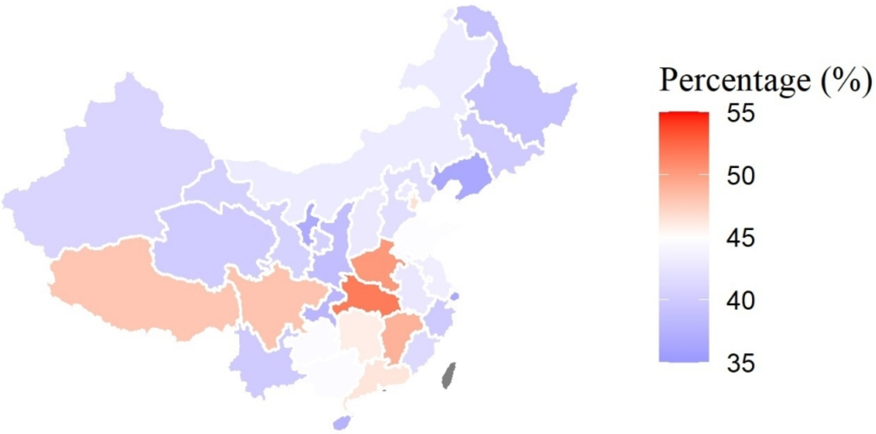

Figure 1. Proportion of the population reporting depression (PHQ-9 >4) by province* (10,000 participants, May 8-June 8, 2020)

121x87mm (300 x 300 DPI)

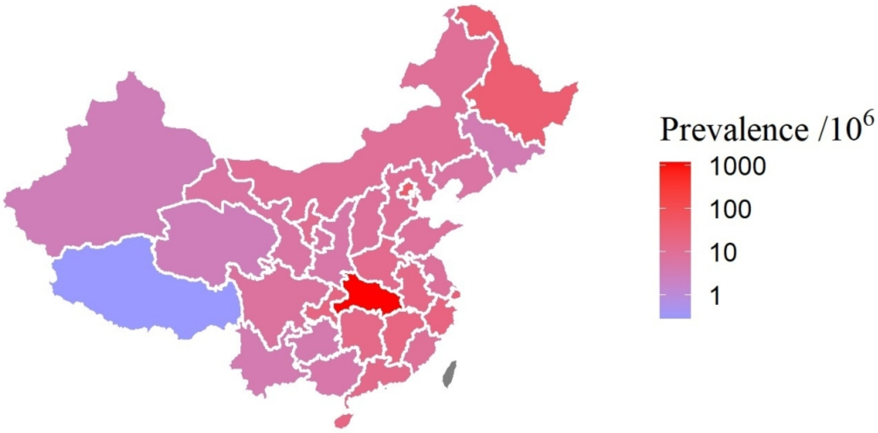

Figure 2. Prevalence of COVID-19 by province* (January 1-June 8, 2020)

121x87mm (300 x 300 DPI)

STROBE Statement—Checklist of items that should be included in reports of ***cross-sectional studies***

	Item No	Recommendation	Page No
**Title and abstract**	1	(*a*) Indicate the study's design with a commonly used term in the title or the abstract	3
		(*b*) Provide in the abstract an informative and balanced summary of what was done and what was found	4
**Introduction**			
Background/rationale	2	Explain the scientific background and rationale for the investigation being reported	5
Objectives	3	State specific objectives, including any prespecified hypotheses	6
**Methods**			
Study design	4	Present key elements of study design early in the paper	7
Setting	5	Describe the setting, locations, and relevant dates, including periods of recruitment, exposure, follow-up, and data collection	7
Participants	6	(*a*) Give the eligibility criteria, and the sources and methods of selection of participants	7
Variables	7	Clearly define all outcomes, exposures, predictors, potential confounders, and effect modifiers. Give diagnostic criteria, if applicable	7
Data sources/ measurement	8*	For each variable of interest, give sources of data and details of methods of assessment (measurement). Describe comparability of assessment methods if there is more than one group	7
Bias	9	Describe any efforts to address potential sources of bias	8
Study size	10	Explain how the study size was arrived at	
Quantitative variables	11	Explain how quantitative variables were handled in the analyses. If applicable, describe which groupings were chosen and why	8
Statistical methods	12	(*a*) Describe all statistical methods, including those used to control for confounding	8
		(*b*) Describe any methods used to examine subgroups and interactions	8
		(*c*) Explain how missing data were addressed	8
		(*d*) If applicable, describe analytical methods taking account of sampling strategy	8
		(*e*) Describe any sensitivity analyses	8
**Results**			
Participants	13*	(a) Report numbers of individuals at each stage of study—eg numbers potentially eligible, examined for eligibility, confirmed eligible, included in the study, completing follow-up, and analysed	9
		(b) Give reasons for non-participation at each stage	9
		(c) Consider use of a flow diagram	9
Descriptive data	14*	(a) Give characteristics of study participants (eg demographic, clinical, social) and information on exposures and potential confounders	9
		(b) Indicate number of participants with missing data for each variable of interest	9
Outcome data	15*	Report numbers of outcome events or summary measures	9
Main results	16	(*a*) Give unadjusted estimates and, if applicable, confounder-adjusted estimates and their precision (eg, 95% confidence interval). Make clear which confounders were adjusted for and why they were included	9

For peer review only - http://bmjopen.bmj.com/site/about/guidelines.xhtml

		(*b*) Report category boundaries when continuous variables were categorized	10
		(*c*) If relevant, consider translating estimates of relative risk into absolute risk for a meaningful time period	10
Other analyses	17	Report other analyses done—eg analyses of subgroups and interactions, and sensitivity analyses	10
**Discussion**			
Key results	18	Summarise key results with reference to study objectives	14
Limitations	19	Discuss limitations of the study, taking into account sources of potential bias or imprecision. Discuss both direction and magnitude of any potential bias	17
Interpretation	20	Give a cautious overall interpretation of results considering objectives, limitations, multiplicity of analyses, results from similar studies, and other relevant evidence	18
Generalisability	21	Discuss the generalisability (external validity) of the study results	18
**Other information**			
Funding	22	Give the source of funding and the role of the funders for the present study and, if applicable, for the original study on which the present article is based	18

*Give information separately for exposed and unexposed groups.

Note: An Explanation and Elaboration article discusses each checklist item and gives methodological background and published examples of transparent reporting. The STROBE checklist is best used in conjunction with this article (freely available on the Web sites of PLoS Medicine at http://www.plosmedicine.org/, Annals of Internal Medicine at http://www.annals.org/, and Epidemiology at http://www.epidem.com/). Information on the STROBE Initiative is available at www.strobe-statement.org.

For peer review only

BMJ Open

The prevalence of depression in China during the early stage of the COVID-19 pandemic: A cross-sectional study in an online survey sample

Journal:	*BMJ Open*
Manuscript ID	bmjopen-2021-056667.R1
Article Type:	Original research
Date Submitted by the Author:	18-Jan-2022
Complete List of Authors:	Liu, Weina; Universitatsklinikum Heidelberg Institut fur Global Health; Jiangsu Provincial CDC,<br>Yu, Fengyun; Tsinghua University Department of Industrial Engineering; Universitatsklinikum Heidelberg Institut fur Global Health<br>Geldsetzer, Pascal; Universitatsklinikum Heidelberg Institut fur Global Health; Stanford University Department of Medicine<br>Yang, Juntao; Chinese Academy of Medical Sciences & Peking Union Medical College<br>Wang, Zhuoran; Chinese Academy of Medical Sciences & Peking Union Medical College<br>Golden, Todd; National Cancer Institute Division of Cancer Control and Population Sciences<br>Jiao, Lirui; Columbia University<br>Chen, Qiushi; The Pennsylvania State University, Harold and Inge Marcus Department of Industrial and Manufacturing Engineering<br>Liu, Haitao; Chinese Academy of Medical Sciences & Peking Union Medical College<br>Wu, Peixin; Chinese Academy of Medical Sciences & Peking Union Medical College<br>Wang, Chen; Chinese Academy of Medical Sciences & Peking Union Medical College; China-Japan Friendship Hospital<br>Bärnighausen, Till ; Universitatsklinikum Heidelberg Institut fur Global Health, Institute of Public Health; Harvard University Department of Global Health and Population<br>Chen , Simiao ; Universitatsklinikum Heidelberg Institut fur Global Health; Chinese Academy of Medical Sciences & Peking Union Medical College
**Primary Subject Heading**:	Public health
Secondary Subject Heading:	Mental health, Public health, Epidemiology
Keywords:	COVID-19, Epidemiology < TROPICAL MEDICINE, MENTAL HEALTH

I, the Submitting Author has the right to grant and does grant on behalf of all authors of the Work (as defined in the below author licence), an exclusive licence and/or a non-exclusive licence for contributions from authors who are: i) UK Crown employees; ii) where BMJ has agreed a CC-BY licence shall apply, and/or iii) in accordance with the terms applicable for US Federal Government officers or employees acting as part of their official duties; on a worldwide, perpetual, irrevocable, royalty-free basis to BMJ Publishing Group Ltd ("BMJ") its licensees and where the relevant Journal is co-owned by BMJ to the co-owners of the Journal, to publish the Work in this journal and any other BMJ products and to exploit all rights, as set out in our licence.

The Submitting Author accepts and understands that any supply made under these terms is made by BMJ to the Submitting Author unless you are acting as an employee on behalf of your employer or a postgraduate student of an affiliated institution which is paying any applicable article publishing charge ("APC") for Open Access articles. Where the Submitting Author wishes to make the Work available on an Open Access basis (and intends to pay the relevant APC), the terms of reuse of such Open Access shall be governed by a Creative Commons licence – details of these licences and which Creative Commons licence will apply to this Work are set out in our licence referred to above.

Other than as permitted in any relevant BMJ Author's Self Archiving Policies, I confirm this Work has not been accepted for publication elsewhere, is not being considered for publication elsewhere and does not duplicate material already published. I confirm all authors consent to publication of this Work and authorise the granting of this licence.

For peer review only

The prevalence of depression in China during the early stage of the COVID-19 pandemic: A cross-sectional study in an online survey sample

Weina Liu[1,2*], Fengyun Yu[1,3,*], Pascal Geldsetzer[1,4,*], Juntao Yang[5], Zhuoran Wang[6], Todd Golden[7], Lirui Jiao[8], Qiushi Chen[9], Haitao Liu[6], Peixin Wu[6,10], Chen Wang[6,11,12,†], Till Bärnighausen[1,6,13,#] , Simiao Chen[1,6,#,†],

[1] Heidelberg Institute of Global Health (HIGH), Faculty of Medicine and University Hospital, Heidelberg University, Heidelberg, Germany

[2] Jiangsu Province Center for Disease Control and Prevention, Nanjing, China

[3] Department of Industrial Engineering, Tsinghua University, Beijing, China

[4] Division of Primary Care and Population Health, Department of Medicine, Stanford University,-Stanford, CA, USA

[5] State Key Laboratory of Medical Molecular Biology, Institute of Basic Medical Sciences, Chinese Academy of Medical Sciences & Peking Union Medical College, Beijing, China

[6] Chinese Academy of Medical Sciences and Peking Union Medical College, Beijing, China

[7] Division of Cancer Control and Population Sciences, National Cancer Institute, Rockville, MD, USA

[8] Columbia University, New York, USA

[9] The Harold and Inge Marcus Department of Industrial and Manufacturing Engineering, The Pennsylvania State University, University Park, PA, USA

[10] Peking Union Medical College Hospital, Beijing, China

[11] National Clinical Research Center for Respiratory Diseases, Beijing, China

[12] Department of Pulmonary and Critical Care Medicine, Center of Respiratory Medicine, China–Japan Friendship Hospital, Beijing, China

[13] Department of Global Health and Population, Harvard T.H. Chan School of Public Health, Boston, MA, USA

For peer review only - http://bmjopen.bmj.com/site/about/guidelines.xhtml

* Co-first authors: WL, FY and PG contributed equally to this study

# Co-senior authors: TB and SC

† Co-corresponding authors:

Simiao Chen, Email: simiao.chen@uni-heidelberg.de

Chen Wang, Email: wangchen@pumc.edu.cn

For peer review only

For peer review only - http://bmjopen.bmj.com/site/about/guidelines.xhtml

Abstract

Objectives: We aimed to determine i) the prevalence of depression during the COVID-19 pandemic among Chinese adults, and ii) how depression prevalence varied by province and sociodemographic characteristics.

Design: Cross-sectional study.

Setting: National online survey in China.

Participants: We conducted a cross-sectional online survey among adults registered with the survey company KuRunData from May 8, 2020 to June 8, 2020. We aimed to recruit 300-360 adults per province (n=14,493), with a similar distribution by sex and rural-urban residency as the general population within each of these provinces.

Primary outcome: Participants completed the Patient Health Questionaire-9 (PHQ-9). We calculated the prevalence of depression (defined as a PHQ-9 score ≥ 10) nationally and separately for each province.

Analysis: Covariate-unadjusted and covariate-adjusted logistic regression models were used to examine how the prevalence of depression varied by adults' sociodemographic characteristics. All analyses used survey sampling weights.

Results: The survey was initiated by 14,493 participants, with 10,000 completing all survey questions and included in the analysis. The prevalence of depression in the national sample was 6.3% (95% CI: 5.7%-6.8%). A higher odds of depression was associated with living in urban area (OR, 1.50; 95% CI, 1.18 – 1.90) and working as a nurse (OR, 3.06; 95% CI, 1.41-6.66). A lower odds of depression was associated with participants who had accurate knowledge of COVID-19 transmission prevention actions (OR, 0.71; 95% CI, 0.51-0.98), the knowledge that saliva is a main transmission route (OR, 0.80; 95% CI, 0.64-0.99), and awareness of COVID-19 symptoms (OR, 0.82; 95% CI, 0.68 – 1.00).

For peer review only - http://bmjopen.bmj.com/site/about/guidelines.xhtml

Conclusion: Around 6.3% adults in our online survey sample had a PHQ-9 score suggestive of depression. Interventions and policies to prevent and treat depression during the COVID-19 pandemic in China may be particularly needed for nurses and those living in urban areas.

Strength and limitation of this study

By sampling adults from each of China's provinces, this is the first study able to assess how the prevalence of depression during the COVID-19 pandemic varied across provinces.

We explored how the prevalence of depression varied within provinces by individuals' socioeconomic characteristics, and are thus able to inform which population groups might be suitable target groups for prevention and treatment of depression during the pandemic.

Our sample is unlikely to be representative of the general population of China because participants had to be registered with an online survey company (KuRunData) to be eligible for the survey.

The study was conducted from May 8, 2020 to June 8, 2020, and thus our findings may not apply to other periods during the pandemic.

For peer review only

Introduction

As of middle January 2022 the World Health Organization (WHO) reports >326 million confirmed cases and >5.53 million deaths worldwide from the coronavirus disease 2019 (COVID-19) pandemic[1]. Effective vaccines are critical to the containment of the COVID-19 pandemic; therefore, more and more vaccines are in clinical and pre-clinical development. Over 56 confirmed effective candidate vaccines for COVID-19 are being produced in America, Europe, China, and Australasia. As of middle January 2022, 139 vaccines are in clinical development, and 194 vaccines are in preclinical development preclinical phases[2]. Moreover, about 59.9% of the world population has uptake at least one dose of COVID-19 vaccine, and 9.68 billion doses have been administered worldwide, as well as 32.92 million doses are administered each day[3]. Although China is currently ranked as one of the countries with the most stringent restrictions, scoring the sixth highest in Stringency Index globally[4], asymptomatic infection and mutated variants continue to pose an unprecedented threat. To reduce the impact of the pandemic, many countries imposed measures including city lockdowns, border closures, social isolation, and quarantine. Long-term lockdowns and a large number of cases may affect not only social and economic outcomes[5-10] but also disrupt normal life leading to pandemic-induced impairments in work and social functioning, including depression [11] even suicide[12].

Previous research suggests that mental health services are crucial to alleviate pandemic-related mental health impacts such as depression[12]. It is essential to understand the geographic distribution of depression to target mental health services on a national, regional, or local level. Non-pharmaceutical policies vary among countries, and they change dynamically as does the prevalence of depression. In China, the estimated overall prevalence of depression was 26.9%[13]. In all studies on depression during the initial COVID-19 pandemic, only four studies included

For peer review only - http://bmjopen.bmj.com/site/about/guidelines.xhtml

the general population[14-18], and none investigated the prevalence of depression by province. Moreover, several cross-sectional studies in different countries suggest an association between depression and sociodemographic characteristics such as female[16], nurses[15], low income[15 19], young age[14 16], lower education level[20], having higher COVID-19 contraction risk, living in urban areas, and social isolation[21]. More recently, literature has emerged contradictory findings about the association between depression and these sociodemographic characteristics[22]. The prevalence of depression across China and the associated sociodemographic characteristics during the COVID-19 pandemic have yet to be fully understood.

Other factors such as whether getting infected with COVID-19, or knowledge and perceptions about the virus, may also have an impact on psychological well-being and mental health. A previous study in India showed that inadequate knowledge of COVID-19 was associated with increased mental health problems [23]. Another study found the perception of COVID-19 may predict psychological well-being through the adoption of different types of coping strategies such as problem-focused coping and emotion-focused coping[24]. Increased knowledge regarding COVID-19 may contribute to better coping strategies, affecting mental health [25].

This study aimed to determine how the prevalence of depression varies by region, sociodemographic characteristics, and other important factors that could affect psychological well-being and mental health, such as previous infections and knowledge and perceptions about the virus. These findings may help formulate effective interventions for improving mental health during different periods of the COVID-19 pandemic in different regions.

For peer review only - http://bmjopen.bmj.com/site/about/guidelines.xhtml

Methods

Study Design and Sampling

We conducted a cross-sectional online survey from May 8, 2020 to June 8, 2020. The survey was carried out with Kurun Information Technology Co. Ltd. (KuRunData, Shanghai, China). The KurunData is an online internet panel for market research, which recruits participants and provides access to the questionnaire via Wechat mini. To assure the representativeness of the data, we utilized stratified sampling by sex, residence type and province. We calculated the size of each stratum based on a sequential method (Table S1) to reflect the province's population composition by sex and urban and rural residence (according to the China Statistical Yearbook 2020 [26]). When sufficient participants were recruited for a given stratum, we halted recruitment for this stratum. Participants were paid 5 yuan (US$0.77) for completing the questionnaire. All participants completed informed written consent. Finally, 14493 participants initiated the online survey, with 10000 participants completing the survey.

Patient and Public Involvement

None of the participants was involved in the design of the questionnaire, nor the design, recruitment, and implementation of the study. We plan to disseminate our study widely through the media, including in China.

Measurements and Covariates

As a part of investigation for willingness to participate in COVID-19 vaccination research, the questionnaire of our study included three parts:1) COVID-19 awareness (perceived risk of death from COVID-19, knowledge of the transmission of COVID-19, awareness of recommended healthcare-seeking behavior); 2) sociodemographic information (sex, age, highest education level, ethnicity, residence type, health care providers, annual household income, personal history of COVID-19 diagnosis , positive acquaintance COVID-19 diagnosis); and 3) the Patient Health Questionnaire-9 (PHQ-9; a depression screening tool [27]).

For peer review only - http://bmjopen.bmj.com/site/about/guidelines.xhtml

We utilized the PHQ-9 depression scale as a screening tool. There are several reasons for using this tool. At only 9 items, it is suitable for a large-scale population survey and easy to be completed by the general population as a quick depression assessment. The nine diagnostic symptom criteria of the PHQ-9 correspond to the DSM-IV major depressive disorder (MDD) criteria and can facilitate follow-up review of symptoms and the diagnosis process[28]. The PHQ-9 was also selected as it has good internal consistency[29 30], reliability, and validity[31 32], with a Cronbach's alpha coefficient between 0.80 and 0.90. We defined a score of 10 or greater as depression; COVID-19 awareness consisted of nine items which were previously published[33]. Among these nine items, for categorical outcomes, data are expressed as binary or categorical (range: 0 -100%). For continuous outcomes, data are expressed as median (interquartile range).

Statistical Analysis

We excluded participants who answered less than half of the questions. All analyses applied sampling weights after excluding incomplete questionnaires. The sampling weights were calculated from the 2019 population census and the sampling quotas, accounting for some feature of the survey, including oversampling for sex, residence type and province. Specifically, sampling weights are the inverse of the probability of selecting participants with some specific residence type (urban or rural), sex (male or female), in some specfic province among the population. We used sampling weights adjusted for the survey design to calculate statistics. For binary and categorical response options of knowledge about COVID-19, we calculated the percentage of participants with correct responses. For binomial proportions, we produced 95% confidence intervals by the Wilson score interval. The prevalence of depression was stratified by participants' sociodemographic characteristics and overall knowledge about COVID-19. To examine how depression prevalence varied by sociodemographic characteristics, we used covariate-unadjusted and covariate-adjusted logistic regression with a binary indicator for each

For peer review only - http://bmjopen.bmj.com/site/about/guidelines.xhtml

province (province-level fixed effects) and obtained odds ratios (OR). All analyses were conducted using R version 4.0.3 (R Foundation for Statistical Computing, Vienna, Austria).

Results

Sociodemographic characteristics

The online survey was initiated by 14,493 participants, with 10,000 completing all survey questions and taking more than two minutes. 4,493 participants were excluded because they took less than two minutes to complete the survey, which we considered insufficient to read and answer all questions.

The majority of participants were male (50.8%), had at least a high school education (73.0%), lived in urban areas (59.4%), and had an annual household income ≥90,000 RMB (US $13,000) (51.6%). Han ethnicity accounted for the vast majority of participants (93.8%). Except for 9% of participants aged under 20 years, the percentage of other age groups ranged from 16.5% to 19.0%. Health care providers accounted for 4.1% of participants, with the largest category being community health workers (1.6%). 0.08% of participants had been diagnosed with COVID-19, and 0.34% of participants knew someone diagnosed with COVID-19. In addition, 6.0% of participants reported being previously diagnosed with depression. The correct rate of COVID-19 awareness ranged from 11.1% to 96.3%, knowledge of COVID-19 transmission prevention actions was lowest (11.1% [95% CI: 10.5% - 11.7%]) and knowledge about the perceived risk of COVID-19 death among people with other diseases was highest (96.3% [95% CI: 96.0% - 96.7%]) (See Table 1.)

For peer review only - http://bmjopen.bmj.com/site/about/guidelines.xhtml

Variation of depression prevalence by province

Overall, the depression prevalence was 6.3% (5.7%-6.8%). As seen in Figure 1, the prevalence of depression in Henan was the highest (9.4% [6.6%-12.7%]) and in Hainan was the lowest (3.7% [1.9%-6.1%]). The prevalence of COVID-19 in Hubei province (1,148 per million) tended to be higher than other areas in China, and Tibet (0.28 per million) saw the lowest prevalence (Table S2). While the prevalence of COVID-19 in other provinces tended to be similar, Hubei and Tibet were exceptions (Figure 2).

Variation of depression prevalence by sociodemographic characteristics within provinces

The results of covariate-unadjusted and covariate-adjusted logistic regression analysis of associated factors with depression are shown in Table 2. After adjusting for covariates, urban residents and nurses had higher odds of depression than rural residents (OR, 1,50; 95% CI, 1.18 – 1.90) and other health care providers (OR, 3.06; 95% CI, 1.41-6.66). Participants who had correct knowledge of COVID-19 transmission prevention actions (OR, 0.71; 95% CI, 0.51 – 0.98) and knowledge of saliva as the main transmission route (OR, 0.80; 95% CI, 0.64 – 0.99) had lower odds of depression, as did participants who had accurate awareness of COVID-19 symptoms (OR, 0.82; 95% CI, 0.68 – 1.00).

Table 1. Sociodemographic Characteristics, COVID-19 Awareness, and Depression Score (PHQ-9) (10,000 survey participants)

Characteristic	Not Depressed (%) (Weighted; n=9396)	Depressed (%) (Weighted; n=604)	Sample characteristics		
			Proportion (Weighted)[1]	N (%) (Unweighted)	Proportion (Unweighted)[2]
Sex					
Female	49.1%	46.2%	48.9%	4,921 (49.2%)	48.9%
Age Group					
< 20 Years	10.5%	11.0%	10.5%	900 (9.0%)	6.9%
20-29 Years	17.1%	17.9%	17.1%	1,645 (16.5%)	20.8%
30-39 Years	18.1%	15.7%	17.9%	1,895 (19.0%)	18.2%
40-49 Years	18.7%	20.5%	18.9%	1,890 (18.9%)	22.1%
50-59 Years	17.9%	20.1%	18.0%	1,820 (18.2%)	16.5%
≥ 60 Years	17.7%	14.9%	17.5%	1,675 (16.8%)	15.4%
Highest Education Level					
less than high school	24.8%	25.5%	24.8%	2792 (27.0%)	68.5%
High School / Technical Secondary School	37.1%	36.8%	37.1%	3,733 (37.3%)	17.6%
College / Undergraduate	36.0%	35.7%	36.0%	3,369 (33.7%)	13.4%

For peer review only

Graduate school and above	2.1%	2.0%	2.1%	206 (2.0%)	0.6%
Ethnicity					
Han	95.1%	94.8%	95.1%	9,381 (93.8%)	95.0%
Man	0.4%	0.6%	0.5%	149 (1.5%)	0.7%
Hui	0.1%	0.2%	0.1%	109 (1.1%)	0.8%
Zang	1.8%	1.5%	1.8%	103 (1.0%)	0.5%
Zhuang	1.4%	1.5%	1.4%	152 (1.5%)	1.2%
Other	1.1%	1.5%	1.1%	106 (1.1%)	1.8%
Province of Residence					
Anhui	4.6%	4.0%	4.5%	360 (3.6%)	4.5%
Beijing	1.5%	1.9%	1.5%	360 (3.6%)	1.5%
Chongqing	2.2%	2.4%	2.2%	360 (3.6%)	2.2%
Fujian	2.8%	2.9%	2.8%	300 (3.0%)	2.8%
Gansu	1.9%	1.8%	1.9%	300 (3.0%)	1.9%
Guangdong	8.1%	9.5%	8.2%	360 (3.6%)	8.2%
Guangxi	3.5%	3.8%	3.5%	300 (3.0%)	3.5%
Guizhou	2.6%	1.8%	2.6%	300 (3.0%)	2.6%
Hainan	0.7%	0.4%	0.7%	300 (3.0%)	0.7%
Hebei	5.4%	5.5%	5.4%	360 (3.6%)	5.4%
Heilongjiang	2.7%	2.1%	2.7%	300 (3.0%)	2.7%
Henan	6.6%	10.3%	6.9%	360 (3.6%)	6.9%
Hubei	4.2%	4.3%	4.2%	360 (3.6%)	4.2%
Hunan	4.9%	4.7%	4.9%	300 (3.0%)	4.9%
Jiangsu	5.7%	7.1%	5.7%	360 (3.6%)	5.7%
Jiangxi	3.4%	2.8%	3.3%	300 (3.0%)	3.3%
Jilin	1.9%	1.8%	1.9%	300 (3.0%)	1.9%
Liaoning	3.1%	2.6%	3.1%	340 (3.4%)	3.1%
Neimengol	1.9%	1.2%	1.8%	300 (3.0%)	1.8%
Ningxia	0.5%	0.4%	0.5%	300 (3.0%)	0.5%
Qinghai	0.4%	0.3%	0.4%	300 (3.0%)	0.4%
Shaanxi	2.8%	2.6%	2.7%	360 (3.6%)	2.7%
Shandong	7.3%	5.7%	7.2%	360 (3.6%)	7.2%
Shanghai	1.7%	1.4%	1.7%	300 (3.0%)	1.7%
Shanxi	2.7%	1.7%	2.8%	300 (3.0%)	2.8%
Sichuan	5.9%	7.1%	6.0%	360 (3.6%)	6.0%
Tianjin	1.1%	1.0%	1.1%	360 (3.6%)	1.1%
Tibet	0.2%	0.3%	0.3%	300 (3.0%)	0.3%
Xinjiang	1.8%	2.2%	1.8%	300 (3.0%)	1.8%
Yunnan	3.5%	2.7%	3.5%	300 (3.0%)	3.5%
Zhejiang	4.2%	3.5%	4.2%	360 (3.6%)	4.2%
Residence Type					
Urban	60.9%	62.9%	61.1%	5,935 (59.4%)	60.6%
Health Care Providers					
No	96.2%	93.6%	96.0%	9,597 (96.0%)	99.0%
Nurse	0.4%	1.3%	0.5%	55 (0.6%)	0.3%
Physician	0.8%	1.3%	0.9%	84 (0.8%)	0.5%
Community Health Worker	1.4%	2.5%	1.5%	157 (1.6%)	<0.1%
Pharmacist	0.1%	0.1%	0.1%	17 (0.2)	<0.1%
Other Healthcare Provider	0.9%	1.2%	1.0%	90 (0.9%)	0.1%
Annual Household Income (RMB)					
< 30,000	5.7%	6.5%	5.8%	560 (5.6%)	
30,000 - 59,999	15.0%	13.9%	14.9%	1,670 (16.7%)	-
60,000 - 89,999	21.7%	19.6%	21.6%	2,303 (23.0%)	-
90,000 - 119,999	25.9%	30.9%	26.2%	2,704 (24.0%)	-
120,000 - 149,999	14.2%	14.9%	14.2%	1,211 (12.1%)	-
150,000 - 199,999	11.1%	8.8%	11.1%	974 (9.7%)	-
≥ 200,000	6.4%	5.4%	6.4%	578(5.8%)	-
Personal COVID-19 Diagnosis History					
No	99.9%	99.8%	99.9%	9,992 (99.9%)	
Yes	0.1%	0.2%	0.1%	8(0.08%)	

For peer review only - http://bmjopen.bmj.com/site/about/guidelines.xhtml

Postive Acquaintance COVID-19 Diagnosis				
Family member	0.1%	0.2%	0.1%	5(0.05%)
Friend	0.2%	0.0%	0.1%	12(0.12%)
Neighbor	0.1%	0.0%	0.1%	7(0.07%)
Cowoker	0.1%	0.3%	0.1%	10(0.1%)
Other	0.0%	0.0%	0.0%	1(0)
COVID-19 Awareness				
Perceived risk of death among vulnerable groups	3.3% (IQR: 1.0%-4.0%)	3.5% (IQR: 1.0%-4.0%)	3.3% (IQR: 1.0%-4.0%)	3.3% (IQR: 1.0%-4.0%)
Perceived risk of death among people with other diseases	96.6% (95% CI: 96.2% - 96.9%)	94.4% (95% CI: 92.2% - 96.0%)	96.4% (95% CI: 96.0% - 96.8%)	96.3% (95% CI: 96.0% - 96.7%)
Perceived elderly as a high-risk group of transmission	92.6% (95% CI: 92.0% - 93.1%)	92.6% (95% CI: 90.1% - 94.4%)	92.6% (95% CI: 92.0% - 93.1%)	92.7% (95% CI: 92.2% - 93.2%)
Awareness of vaccine availability protecting against transmission	76.7% (95% CI: 75.8% - 77.5%)	74.9% (95% CI: 71.2% - 78.1%)	76.5% (95% CI: 75.7% - 77.3%)	76.0% (95% CI: 75.1% - 76.8%)
Awareness that masks are highly effective in protecting against transmission	89.3% (95% CI: 88.7% - 89.9%)	88.0% (95% CI: 85.1% - 90.4%)	89.2% (95% CI: 88.6% - 89.8%)	89.8% (95% CI: 89.2% - 90.4%)
Knowledge of transmission prevention actions	11.6% (95% CI: 10.9% - 12.2%)	8.1% (95% CI: 6.2% - 10.6%)	11.4% (95% CI: 10.8% - 12.0%)	11.1% (95% CI: 10.5% - 11.7%)
Knowledge of saliva as main transmission route	82.6% (95% CI: 81.8% - 83.3%)	76.9% (95% CI: 73.4% - 80.1%)	82.2% (95% CI: 81.5% - 83.0%)	82.1% (95% CI: 81.3% - 82.8%)
Awareness of COVID-19 symptoms	69.4% (95% CI: 68.5% - 70.3%)	64.4% (95% CI: 60.5% - 68.1%)	69.1% (95% CI: 68.2% - 70.0%)	67.1% (95% CI: 66.1% - 68.0%)
Awareness of recommended healthcare-seeking behavior	71.6% (95% CI: 70.7% - 72.5%)	68.2% (95% CI: 64.3% - 71.8%)	71.4% (95% CI: 70.5% - 72.3%)	68.8% (95% CI: 67.9% - 69.7%)

[1] Weighted using survey sampling weights.

[2] As per the 2020 China Statistical Yearbook.

For dichotomous outcomes, data are expressed as a percentage with the correct response (95% confidence interval). For continuous outcomes, data are expressed as median (interquartile range).

Table 2. Covariate-unadjusted and covariate-adjusted logistic regressions of depression onto sociodemographic characteristics

	**Covariate-unadjusted OR (95% CI)** [1]	**Covariate-adjusted OR (95% CI)**[2]	**Depression prevalence (95% CI)**
Overall			604 (6.3%)[5.7%~6.8%]
Sex			
Male	Ref.	Ref.	323 (6.6%)[5.8%~7.4%]
Female	0.90 (0.75 - 1.07)	0.89 (0.74 - 1.07)	281 (5.9%)[5.2%~6.7%]
Age			
18-19 years	Ref.	Ref.	65 (6.5%)[4.9%~8.4%]
20-29 years	1.01 (0.72 - 1.42)	1.06 (0.72 - 1.56)	117 (6.5%)[5.2%~8.0%]
30-39 years	0.85 (0.59 - 1.21)	0.91 (0.60 - 1.37)	98 (5.5%)[4.3%~6.8%]
40-49 years	1.07 (0.76 - 1.49)	1.12 (0.77 - 1.63)	114 (6.8%)[5.5%~8.3%]
50-59 years	1.09 (0.78 - 1.53)	1.05 (0.72 - 1.54)	120 (7.0%)[5.7%~8.4%]
>60 years	0.83 (0.58 - 1.19)	0.82 (0.52 - 1.31)	90 (5.3%)[4.2%~6.6%]
Highest Education Level			
less than high school	Ref	Ref	165 (7.2%)[5.8%~8.7%]
High school / Technical secondary school	1.48 (0.85 - 2.59)	1.16 (0.61 - 2.21)	225 (6.2%)[5.3%~7.2%]
College / Undergraduate	1.45 (0.83 - 2.54)	1.12 (0.58 - 2.14)	202 (6.2%)[5.3%~7.2%]
Graduate school and above	1.37 (0.58 - 3.25)	1.09 (0.43 - 2.75)	12 (5.9%)[2.6%~10.5%]
Residence Type			
Rural	Ref.	Ref.	243 (6.0%)[5.1%~6.9%]

For peer review only - http://bmjopen.bmj.com/site/about/guidelines.xhtml

Urban	1.09 (0.90 - 1.31)	1.50 (1.18 - 1.90)**	361 (6.5%)[5.8%~7.2%]
Health Care Providers			
No	Ref.	Ref.	564 (6.1%)[5.6%~6.7%]
Nurse	2.61 (1.18 - 5.75)*	3.06 (1.41 - 6.66)**	7 (16.1%)[5.1%~31.5%]
Physician	1.48 (0.65 - 3.38)	1.51 (0.67 - 3.40)	7 (9.2%)[2.9%~18.5%]
Community health worker	1.66 (0.97 - 2.86)	1.62 (0.94 - 2.78)	16 (10.4%)[5.5%~16.6%]
Pharmacist	0.91 (0.18 - 4.71)	1.17 (0.24 - 5.69)	2 (11.8%)[NA~NA]
Other healthcare provider	1.38 (0.65 - 2.92)	1.31 (0.60 - 2.90)	8 (8.1%)[2.9%~15.5%]
Annual household income (RMB)			
< 30,000	Ref.	Ref.	39 (7.1%)[4.8%~9.8%]
30,000 – 59,999	0.87 (0.58 - 1.31)	0.92 (0.60 - 1.42)	98 (5.8%)[4.6%~7.2%]
60,000 – 89,999	0.82 (0.55 - 1.22)	0.87 (0.56 - 1.34)	132 (5.7%)[4.7%~6.9%]
90,000 – 119,999	1.05 (0.71 - 1.56)	1.14 (0.74 - 1.75)	182 (7.4%)[6.3%~8.7%]
120,000 – 149,999	0.87 (0.56 - 1.34)	0.92 (0.56 - 1.49)	78 (6.6%)[5.1%~8.2%]
150,000 – 199,999	0.68 (0.42 - 1.10)	0.70 (0.41 - 1.20)	44 (5.0%)[3.6%~6.7%]
≥ 200,000	0.69 (0.40 - 1.20)	0.72 (0.39 - 1.32)	31 (5.3%)[3.4%~7.7%]
COVID-19 Awareness			
Perceived risk of death among vulnerable groups	1.02 (0.99 - 1.05)	1.02 (0.99 - 1.05)	NA
Perceived risk of death among people with other diseases	0.87 (0.72 - 1.05)	0.88 (0.73 - 1.07)	395 (6.0%)[5.4%~6.7%]
Perceived elderly as a high-risk group of transmission	1.00 (0.71 - 1.40)	1.05 (0.75 - 1.47)	558 (6.3%)[5.7%~6.9%]
Awareness of vaccine availability protecting against transmission	0.92 (0.75 - 1.13)	0.91 (0.73 - 1.13)	447 (6.1%)[5.5%~6.8%]
Awareness that masks are highly effective in protecting against transmission	0.88 (0.67 - 1.16)	0.89 (0.68 - 1.17)	533 (6.2%)[5.6%~6.8%]
Knowledge of transmission prevention actions	0.68 (0.49 - 0.94)*	0.71 (0.51 - 0.98)*	50 (4.5%)[3.2%~6.0%]
Knowledge of saliva as main transmission route	0.74 (0.60 - 0.91)**	0.80 (0.64 - 0.99)*	464 (5.9%)[5.3%~6.5%]
Awareness of COVID-19 symptoms	0.81 (0.67 - 0.97)*	0.82 (0.68 - 1.00)*	386 (5.8%)[5.2%~6.5%]
Awareness of recommended healthcare - seeking behavior	0.84 (0.69 - 1.02)	0.81 (0.65 - 1.01)	404 (6.0%)[5.4%~6.7%]
Postive Acquaintance COVID-19 Diagnosis			
No	Ref.	Ref.	602 (6.3%)[5.7%~6.8%]
Yes	1.60 (0.44 - 5.84)	1.22 (0.32 - 4.61)	2 (10.0%)[0.5%~29.5%]
COVID-19 cases confirmed by the province			
Prevalence of COVID-19 by province	1.00 (0.99 - 1.01)	1.00 (0.99 - 1.02)	NA

[1]. Covariate-unadjusted logistic regression results [All regressions included each one of the variables shown in the table and adjusted for each province (province-level fixed effects)]

[2]. Covariate-adjusted logistic regression results [All regressions included all variables shown in the table and adjusted for each province (province-level fixed effects)]

***represents *p<0.001;* **represents *p<0.01;* * represents *p<0.05*

Discussion

In our online survey sample, 6.3% of adults had depression as defined by a PHQ-9 ≥10. We found a higher prevalence of depression among certain population groups, including urban residents and nurses. Furthermore, we found that knowledge and awareness of COVID-19 were associated with a lower odds of depression. Under the strong assumption that these associations might be causal, this finding could indicate a "protective effect" for mental health and thus indicate the importance of effective communication and education about COVID-19 amid the pandemic.

The correlation between urban residence and depression was positive in our study, which is consistent with several previous studies [34 35]. A possible interpretation of this finding is that while the virus could be transmitted more quickly in urban areas with a higher density population [36], those in urban areas tend to have higher education levels and greater access to the latest report on the COVID-19 pandemic[34]. Another explanation is that depression was more common in the urban than in rural areas in China before the COVID-19 pandemic. Moreover, the social distancing restrictions allowed less travel in cities than in rural areas, potentially contributing to the higher prevalence of depression in urban than in rural areas. This study also found that nurses had a higher odds of depression, consistent with previous studies[37 38]. One possible explanation was that as front-line healthcare workers, nurses had higher risk to be infected by longer contact with patients than doctors, and worked longer hours than usual[39], which might make them become more frustrated.

Several studies have described the prevalence of depression during the COVID-19 pandemic in the general population. Among studies using the PHQ-9 scale with the same cut-off value(≥

For peer review only - http://bmjopen.bmj.com/site/about/guidelines.xhtml

10), the prevalence of depression observed in our study (6.3%) was lower than a national study among 56,679 participants conducted February 28 to March 11, 2020 in China (10.8%)[40] as well as a study of 1,470 individuals among the general population during the COVID-19 outbreak in the United States from March 31 to April 13, 2020 (27.8%)[41]. Most of the other studies were conducted in February 2020─at the peak of the COVID-19 epidemic, which ranged from 8.3 to 48.3%[40 42-44]. One possible explanation is that our study occurred in May when the epidemic had decreased in severity, and the prevalence of depression may have diminished. Another possible reason for a lower depression prevalence found in our study may be that with the continuous strict quarantine policy, the government provided timely mental health service in response to COVID-19[45].

The prevalence of COVID-19 in 31 provinces tended to be similar except for Hubei, which had the highest prevalence (Figure 2). However, the proportion of the population by province with depression (PHQ-9 $\geq$ 10) did not seem to be significantly associated with the prevalence of COVID-19 cases. Moreover, from the covariate-adjusted logistic regression results, we found that depression was not associated with the prevalence of COVID-19 cases confirmed by province, which also suggested that the prevalence of depression is not associated with the severity of the regional epidemic during the COVID-19 outbreak. One explanation is that resilience plays a protective role in mitigating the impact of stress and trauma on depressive symptoms and the consequences associated with depressive symptoms[46]. During the initial phase of the COVID-19 outbreak in China, the Chinese government enforced a rigid social distancing policy through social media and strongly promoted hand washing, surface disinfection, and the use of protective masks[47]. In Wuhan, as the most affected city, people with mild and asymptomatic infection received care in Fangcang shelter hospitals, for facility-based isolation, treatment, and monitoring [47], an effective method to control the epidemic [48 49].

For peer review only - http://bmjopen.bmj.com/site/about/guidelines.xhtml

Fangcang shelter hospitals also provided mental health counseling services and social support to help patients recover during isolation [50], which may also reduce the incidence of depression in hard-hit areas. Due to very limited confirmed cases in our study, it was difficult to investigate the association between depression and the number of confirmed cases (0.1% participants diagnosed with COVID-19, 0.48% participants with an acquaintance with confirmed COVID-19).

This study, for the first time, provides preliminary evidence that knowledge of COVID-19 transmission prevention actions, knowledge of saliva as main transmission route and awareness of COVID-19 symptoms may be associated with a lower risk of depression. Compared to previous research[34 51 52], Our study reinforced the positive association between precautionary measures as well as awareness of COVID-19 symptoms and depression. One possible explanation could be that accurate knowledge of COVID-19 transmission prevention actions and awareness of COVID-19 symptoms may help alleviate the stress caused by fear of COVID-19 pandemic. Stress, as a risk factor for depression, plays a role in triggering depression by biological mechanisms such as HPA axis stress response processes and hormonal and neurotransmitter systems [53]. Therefore, accurate knowledge and perceptions towards COVID-19 could indirectly affect depression through the reduction in stress. This finding suggests that, to reduce the prevalence of depression, effective communication and education of COVID-19 preventive measures and recommended healthcare-seeking behaviors are urgently needed.

Limitations

This is the first study that investigated the prevalence of depression by province during the early stage of the COVID-19 pandemic in China; however, our study has several limitations. First, although our sample size was large, all participants needed to register with KuRunData therefore our sample is unlikely to be representative of the general population of China. As this

For peer review only - http://bmjopen.bmj.com/site/about/guidelines.xhtml

was an online survey, participants had access to a computer or smartphone and therefore tended to be younger and highly educated. Although we used stratified sampling to increase the representativeness of the data, it is still difficult to avoid response bias as potential participants with depression might be either less or more interested in taking part in the survey. Also, our measure of depression was the PHQ-9 score, which is a tool for screening for depression but is not recommended for a clinical diagnosis of the condition.

For peer review only

Conclusions

In conclusion, the prevalence of depression in our national online survey sample was around six percent during the initial stage of the COVID-19 pandemic. Depression was more common among nurses and in urban areas. Accurate knowledge of COVID-19 transmission and awareness of COVID-19 symptoms were associated with lower odds of depression. These findings may assist with targeted efforts to prevent or treat depression in China to those most in need of mental health services during the COVID-19 pandemic.

Contributors

WL and SC conceived the idea, JY, ZW, HL,PW, collected data, FY and WL analyzed and verified data, WL wrote the first draft, PG and TG edited the manuscript, LJ, QC, SC, CW and TB collaboratively reviewed the manuscript.

Funding

This work was supported by the Bill & Melinda Gates Foundation (Project INV-006261) and the Sino- German Center for Research Promotion (Project C-0048). The funding source had involvement in study design and data collection.

Competing interests

The authors have declared no conflicts of interest.

Patient consent for publication

Not required.

Ethics approval

This research was approved by the institutional review board of Chinese Academy of Medical Sciences & Peking Union Medical College(CAMS&PUMC-IEC-2020-001), and authors had access only to de-identified data.

Data availability statement

The primary dataset reported in this publication will be available but need to be obtained permission from corresponding author Simiao Chen.

References

1. World Health Organization. Coronavirus disease (COVID-19) pandemic [Available from: https://covid19.who.int/?gclid=Cj0KCQjw8IaGBhCHARIsAGIRRYpDW4NS0CUiiW6a4fYWJ-AdgKZk4MDgbs9ck5gmi1c21JXMESfvjZwaAkP4EALw_wcB accessed 18 January 2022.
2. World Health Organization. The COVID-19 vaccine tracker and landscape compiles detailed information of each COVID-19 vaccine candidate in development by closely monitoring their progress through the pipeline 2022 [Available from: https://www.who.int/publications/m/item/draft-landscape-of-covid-19-candidate-vaccines.
3. Hannah Ritchie EM, Lucas Rodés-Guirao, Cameron Appel, Charlie Giattino, Esteban Ortiz-Ospina, Joe Hasell, Bobbie Macdonald, Diana Beltekian and Max Roser. Coronavirus Pandemic (COVID-19): Our World in Data; 2022 [Available from: https://ourworldindata.org/coronavirus.
4. Tracker OC-GR. Government Response Stringency Index. *Our World in Data*
5. Wang C, Horby PW, Hayden FG, et al. A novel coronavirus outbreak of global health concern. *The lancet* 2020;395(10223):470-73.
6. Roberton T, Carter ED, Chou VB, et al. Early estimates of the indirect effects of the COVID-19 pandemic on maternal and child mortality in low-income and middle-income countries: a modelling study. *The Lancet Global Health* 2020;8(7):e901-e08.
7. Cutler DM, Summers LH. The COVID-19 pandemic and the $16 trillion virus. *Jama* 2020;324(15):1495-96.
8. Do D, Sarker M, Chen S, et al. Healthcare worker attendance during the early stages of the COVID-19 pandemic: A longitudinal analysis of fingerprint-verified data from all public-sector secondary and tertiary care facilities in Bangladesh. *Journal of Global Health* 2020;10(2)
9. Chen S, Prettner K, Kuhn M, et al. The economic burden of COVID-19 in the United States: Estimates and projections under an infection-based herd immunity approach.

For peer review only - http://bmjopen.bmj.com/site/about/guidelines.xhtml

The Journal of the Economics of Ageing 2021:100328. doi: https://doi.org/10.1016/j.jeoa.2021.100328
10. Prettner K, Chen S, Kuhn M, et al. Effective pandemic management that minimises economic harm. *Europe* 2021
11. Bao Y, Sun Y, Meng S, et al. 2019-nCoV epidemic: address mental health care to empower society. *The Lancet* 2020;395(10224):e37-e38.
12. Dawel A, Shou Y, Smithson M, et al. The effect of COVID-19 on mental health and wellbeing in a representative sample of Australian adults. *Frontiers in psychiatry* 2020;11:1026.
13. Bareeqa SB, Ahmed SI, Samar SS, et al. Prevalence of depression, anxiety and stress in china during COVID-19 pandemic: A systematic review with meta-analysis. *The International Journal of Psychiatry in Medicine* 2021;56(4):210-27.
14. Al Zabadi H, Alhroub T, Yaseen N, et al. Assessment of Depression Severity During Coronavirus Disease 2019 Pandemic Among the Palestinian Population: A Growing Concern and an Immediate Consideration. *Frontiers in psychiatry* 2020;11:570065. doi: 10.3389/fpsyt.2020.570065
15. Batra K, Singh TP, Sharma M, et al. Investigating the Psychological Impact of COVID-19 among Healthcare Workers: A Meta-Analysis. *Int J Environ Res Public Health* 2020;17(23) doi: 10.3390/ijerph17239096
16. Hamzeh AZ, Thair AH, Noor Y, et al. Assessment of depression severity during COVID-19 pandemic among the Palestinian population: a growing concern and an immediate consideration. *J Frontiers in psychiatry* 2020;11:1486.
17. Huang Y, Zhao N. Generalized anxiety disorder, depressive symptoms and sleep quality during COVID-19 outbreak in China: a web-based cross-sectional survey. *Psychiatry Res* 2020;288:112954. doi: 10.1016/j.psychres.2020.112954
18. Wang C, Pan R, Wan X, et al. A longitudinal study on the mental health of general population during the COVID-19 epidemic in China. *Brain, behavior, and immunity* 2020;87:40-48. doi: 10.1016/j.bbi.2020.04.028
19. Sazakli E, Leotsinidis M, Bakola M, et al. Prevalence and associated factors of anxiety and depression in students at a Greek university during COVID-19 lockdown. *Journal of public health research* 2021
20. Lei L, Huang X, Zhang S, et al. Comparison of prevalence and associated factors of anxiety and depression among people affected by versus people unaffected by quarantine during the COVID-19 epidemic in Southwestern China. *J Medical science monitor: international medical journal of experimental clinical research* 2020;26:e924609-1.
21. Durankuş F, Aksu E. Effects of the COVID-19 pandemic on anxiety and depressive symptoms in pregnant women: a preliminary study. *The Journal of Maternal-Fetal Neonatal Medicine* 2020:1-7.
22. Zhang W, Wang K, Yin L, et al. Mental health and psychosocial problems of medical health workers during the COVID-19 epidemic in China. *Psychotherapy psychosomatics* 2020;89(4):242-50.
23. Mishra P, Bhadauria US, Dasar PL, et al. Knowledge, attitude and anxiety towards pandemic flu a potential bio weapon among health professionals in Indore City. *Przeglad epidemiologiczny* 2016;70(1):41-5, 125.
24. Krok D, Zarzycka B. Risk Perception of COVID-19, Meaning-Based Resources and Psychological Well-Being amongst Healthcare Personnel: The Mediating Role of Coping. *Journal of clinical medicine* 2020;9(10):3225.
25. Floyd DL, Prentice-Dunn S, Rogers RW. A meta-analysis of research on protection motivation theory. *Journal of applied social psychology* 2000;30(2):407-29.

For peer review only - http://bmjopen.bmj.com/site/about/guidelines.xhtml

26. National Bureau of Statistics of China. The urban-rural composition of the population by region and the birth rate, death rate, and natural growth rate（2019） Beijing2020 [cited 2021 May 30]. Available from: http://www.stats.gov.cn/tjsj/ndsj/2020/indexch.htm accessed May 30 2021.
27. Kroenke K, Spitzer RL. The PHQ-9: A New Depression Diagnostic and Severity Measure. *Psychiatr Ann* 2002;32(9):509-21.
28. Fann JR, Berry DL, Wolpin S, et al. Depression screening using the Patient Health Questionnaire-9 administered on a touch screen computer. *Psycho-Oncology: Journal of the Psychological, Social Behavioral Dimensions of Cancer* 2009;18(1):14-22.
29. Spitzer RL, Kroenke K, Williams JB, et al. Validation and utility of a self-report version of PRIME-MD: the PHQ primary care study. *Jama* 1999;282(18):1737-44.
30. Kroenke K, Spitzer RL, Williams JB. The PHQ-9: validity of a brief depression severity measure. *Journal of general internal medicine* 2001;16(9):606-13.
31. Wang W, Bian Q, Zhao Y, et al. Reliability and validity of the Chinese version of the Patient Health Questionnaire (PHQ-9) in the general population. *General hospital psychiatry* 2014;36(5):539-44.
32. Zhang Y, Liang W, Chen Z, et al. Validity and reliability of P atient H ealth Q uestionnaire-9 and P atient H ealth Q uestionnaire-2 to screen for depression among college students in C hina. *Asia-Pacific Psychiatry* 2013;5(4):268-75.
33. Yu F, Geldsetzer P, Meierkord A, et al. Knowledge About COVID-19 Among Adults in China: Cross-sectional Online Survey. *Journal of Medical Internet Research* 2021;23(4):e26940.
34. Özdin S, Bayrak Özdin Ş. Levels and predictors of anxiety, depression and health anxiety during COVID-19 pandemic in Turkish society: The importance of gender. *International Journal of Social Psychiatry* 2020;66(5):504-11.
35. Chen Y, Jin Y, Zhu L, et al. The network investigation on knowledge, attitude and practice about COVID-19 of the residents in Anhui Province. *Zhonghua yu fang yi xue za zhi* 2020;54(4):367-73.
36. Taylor S. The psychology of pandemics: Preparing for the next global outbreak of infectious disease: Cambridge Scholars Publishing 2019.
37. Cabarkapa S, Nadjidai SE, Murgier J, et al. The psychological impact of COVID-19 and other viral epidemics on frontline healthcare workers and ways to address it: A rapid systematic review. *Brain, behavior, immunity-health* 2020:100144.
38. Sriharan A, West KJ, Almost J, et al. COVID-19-Related Occupational Burnout and Moral Distress among Nurses: A Rapid Scoping Review. *Nursing leadership (Toronto, Ont)* 2021;34(1):7-19. doi: 10.12927/cjnl.2021.26459
39. Lai J, Ma S, Wang Y, et al. Factors associated with mental health outcomes among health care workers exposed to coronavirus disease 2019. *JAMA network open* 2020;3(3):e203976-e76.
40. Gao J, Zheng P, Jia Y, et al. Mental health problems and social media exposure during COVID-19 outbreak. *Plos one* 2020;15(4):e0231924.
41. Shi L, Lu Z, Que J, et al. Prevalence of and risk factors associated with mental health symptoms among the general population in China during the coronavirus disease 2019 pandemic. *JAMA network open* 2020;3(7):e2014053-e53.
42. Qiu J, Shen B, Zhao M, et al. A nationwide survey of psychological distress among Chinese people in the COVID-19 epidemic: implications and policy recommendations. *General psychiatry* 2020;33(2)

For peer review only - http://bmjopen.bmj.com/site/about/guidelines.xhtml

43. Ahmed MZ, Ahmed O, Aibao Z, et al. Epidemic of COVID-19 in China and associated psychological problems. *Asian journal of psychiatry* 2020;51:102092.
44. Zhou S, Zhang L, Wang L, et al. Prevalence and socio-demographic correlates of psychological health problems in Chinese adolescents during the outbreak of COVID-19. *European Child Adolescent Psychiatry* 2020;29(6):749-58.
45. Ju Y, Zhang Y, Wang X, et al. China's mental health support in response to COVID-19: progression, challenges and reflection. *Globalization and Health* 2020;16(1):102. doi: 10.1186/s12992-020-00634-8
46. Bitsika V, Sharpley CF, Bell R. The buffering effect of resilience upon stress, anxiety and depression in parents of a child with an autism spectrum disorder. *Journal of Developmental Physical Disabilities* 2013;25(5):533-43.
47. Chen S, Yang J, Yang W, et al. COVID-19 control in China during mass population movements at New Year. *The Lancet* 2020;395(10226):764-66.
48. Chen S, Chen Q, Yang J, et al. Curbing the COVID-19 pandemic with facility-based isolation of mild cases: a mathematical modeling study. *Journal of Travel Medicine* 2020;28(2) doi: 10.1093/jtm/taaa226
49. Dickens BL, Koo JR, Wilder-Smith A, et al. Institutional, not home-based, isolation could contain the COVID-19 outbreak. *The Lancet* 2020;395(10236):1541-42.
50. Chen S, Zhang Z, Yang J, et al. Fangcang shelter hospitals: a novel concept for responding to public health emergencies. *The Lancet* 2020;395(10232):1305-14.
51. Qian Y, Wu K, Xu H, et al. A survey on physical and mental distress among cancer patients during the COVID-19 epidemic in Wuhan, China. *Journal of palliative medicine* 2020;23(7):888-89.
52. Wang C, Pan R, Wan X, et al. Immediate Psychological Responses and Associated Factors during the Initial Stage of the 2019 Coronavirus Disease (COVID-19) Epidemic among the General Population in China. *Int J Environ Res Public Health* 2020;17(5) doi: 10.3390/ijerph17051729
53. Hammen CL. Stress and depression: old questions, new approaches. *Current Opinion in Psychology* 2015;4:80-85.

For peer review only - http://bmjopen.bmj.com/site/about/guidelines.xhtml

Figure 1. Proportion of the population reporting depression (PHQ-9 ≥10) by province*

(10,000 participants, May 8-June 8, 2020)

* Taiwan, Hong Kong and Macao are shown in grey.

Source of map: http://datav.aliyun.com/portal/school/atlas/area_selector

Figure 2 . Prevalence of COVID-19 by province* (January 1-June 8, 2020)

* Taiwan, Hong Kong and Macao are shown in grey.

Source of map: http://datav.aliyun.com/portal/school/atlas/area_selector

For peer review only

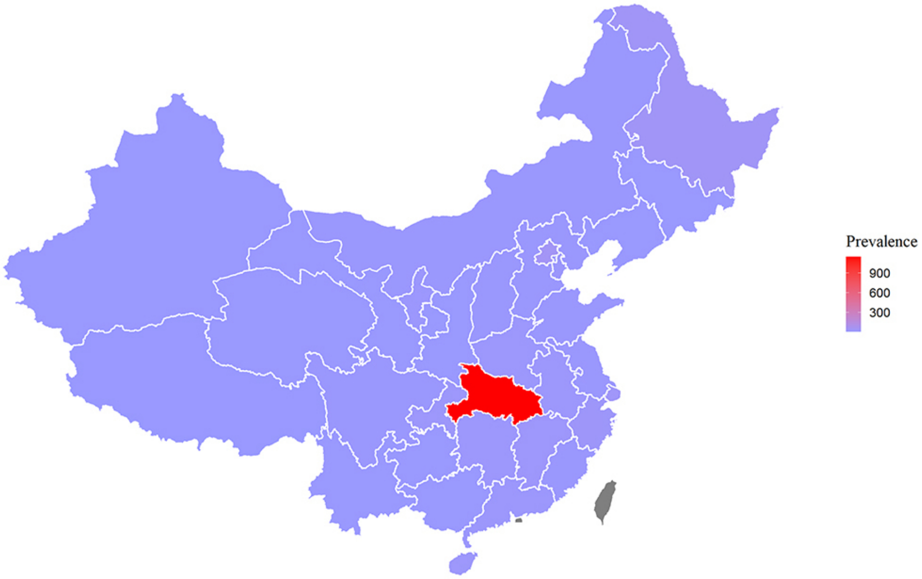

Figure 1. Proportion of the population reporting depression (PHQ-9 ≥10) by province* (10,000 participants, May 8-June 8, 2020)
* Taiwan, Hong Kong and Macao are shown in grey.

90x90mm (300 x 300 DPI)

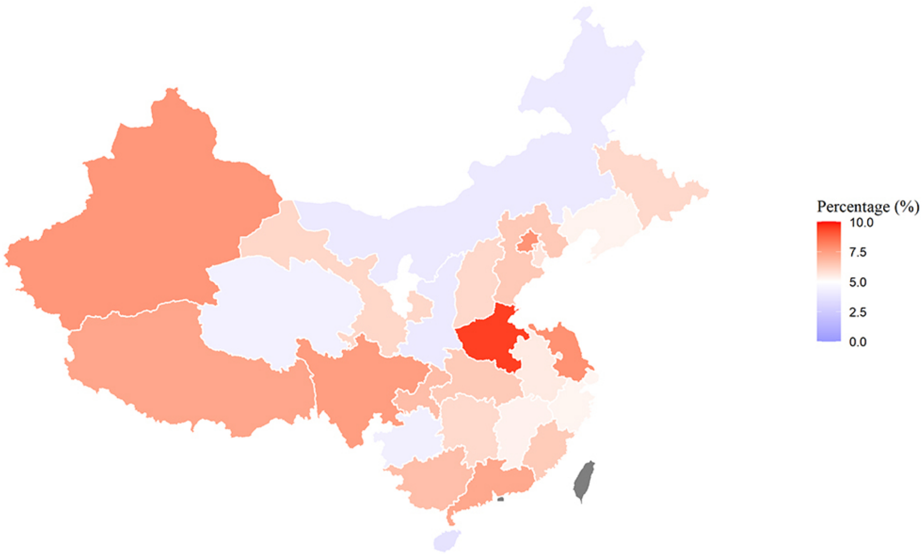

Figure 2 . Prevalence of COVID-19 by province* (January 1-June 8, 2020)
* Taiwan, Hong Kong and Macao are shown in grey.

90x90mm (300 x 300 DPI)

Appendix 1

Sample size calculation with sequential methods were showed in table S1. Firstly, we make each province have 300 quotas; Next, we allocate 360 quotas to provinces that have more than 70 million; After allocating quotas according to each province's urban and sex ratio, we found that the number of the stratum Beijing-rural-male, Beijing-rural-female, Shanghai-rural-male, Shanghai-rural-female, Tianjin-rural-male, Tianjin-rural-female, Guangdong-rural-female were less than 50, so we adjusted the quotas according to the following steps:

1) We raised the quotas for Beijing-rural-male, Beijing-rural-female, Shanghai-rural-male, Shanghai-rural-female, Tianjin-rural-male, Tianjin-rural-female, Guangdong-rural-female to 50.

2) We raised the total quotas for Beijing, Tianjin and Shanghai, Zhejiang, Anhui to 360, total quotas for Liaoning to 340, thus we can achieve a goal with 10000 total sample.

3) We allocated the rest 260 quotas for Beijing-urban-male, Beijing-urban-female, Shanghai-urban-male, Shanghai-urban-female, Tianjin-urban-male, Tianjin-urban-female; and allocate the rest 310 quotas for Guangdong-urban-male, Guangdong-urban-female, Guangdong-rural-male.

Table S1 Sample size calculation with sequential methods of each province in China

City	Population (million)	Sex ratio (female=100)	City %	Rural %	Sample	City-Male	City-Female	Rural-Male	Rural-Female
Beijing	2154	98.75	86.50%	13.50%	360	133	127	50	50
Tianjing	1560	115.72	83.15%	16.85%	360	142	118	50	50
Hebei	7556	101.67	56.43%	43.57%	360	102	103	78	77
Shanxi	3718	104.73	58.41%	41.59%	300	89	87	63	61
Neimenggu	2534	104.26	62.71%	37.29%	300	95	94	57	54
Liaoning	4359	100.37	68.10%	31.90%	340	115	117	54	54
Jiling	2704	102.03	57.53%	42.47%	300	87	87	64	62
Heilongjiang	3773	103.93	60.10%	39.90%	300	91	90	61	58

For peer review only

Shanghai	2424	106.61	88.10%	11.90%	360	135	125	50	50
Jiangsu	8051	103.43	69.61%	30.39%	360	127	125	55	53
Zhejiang	5737	107.97	68.90%	31.10%	360	128	121	58	53
Anhui	6324	106.06	54.69%	45.31%	360	101	97	83	79
Fujian	3941	108.41	65.82%	34.18%	300	102	95	53	50
Jiangxi	4648	105.83	56.02%	43.98%	300	86	83	67	64
Shandong	10047	100.77	61.18%	38.82%	360	110	111	70	69
Henan	9605	102.45	51.71%	48.29%	360	94	94	87	85
Hubei	5917	106.15	60.30%	39.70%	300	92	90	61	57
Hunan	6899	101.51	56.02%	43.98%	300	84	85	66	65
Guangdong	11346	117.27	70.70%	29.30%	360	137	117	56	50
Guangxi	4926	108.47	50.22%	49.78%	300	78	74	77	71
Hainan	934	104.69	59.06%	40.94%	300	90	88	62	60
Chongqing	3102	100.35	65.50%	34.50%	300	98	100	51	51
Sichuan	8341	98.67	52.29%	47.71%	360	93	96	85	86
Guizhou	3600	109.37	47.52%	52.48%	300	74	69	82	75
Yunnan	4830	107.67	47.81%	52.19%	300	74	70	81	75
Xizang	344	98.98	31.14%	68.86%	300	50	50	98	102
Shanxi	3864	100.47	58.13%	41.87%	300	87	89	62	62
Gansu	2637	103.65	47.69%	52.31%	300	72	72	80	76
Qinghai	603	108.34	54.47%	45.53%	300	84	80	71	65
Ningxia	688	98.27	58.88%	41.12%	300	87	90	61	62
Xinjiang	2487	99.76	50.91%	49.09%	300	76	78	73	73
Total					10000				

For peer review only

Table S2 Confirmed cases of each province in China

Province	Confirmed cases
Beijing	587
Shanghai	675
Tianjing	194
Chongqing	573
Liaoning	147
Jiling	153
Heilongjiang	934
Hebei	334
Shanxi	198
Neimenggu	237
Jiangsu	653
Zhejiang	1267
Anhui	985
Fujian	357
Jiangxi	931
Shandong	785
Henan	1254
Hubei	63623
Hunan	1015
Guangdong	1627
Guangxi	252
Hainan	165
Sichuan	578
Guizhou	145
Yunnan	183
Xizang	1
Shanxi	308
Gansu	146
Qinghai	18
Ningxia	75
Xinjiang	73

For peer review only

STROBE Statement—Checklist of items that should be included in reports of ***cross-sectional studies***

	Item No	Recommendation	Page No
**Title and abstract**	1	(*a*) Indicate the study's design with a commonly used term in the title or the abstract	3
		(*b*) Provide in the abstract an informative and balanced summary of what was done and what was found	4
**Introduction**			
Background/rationale	2	Explain the scientific background and rationale for the investigation being reported	5
Objectives	3	State specific objectives, including any prespecified hypotheses	6
**Methods**			
Study design	4	Present key elements of study design early in the paper	7
Setting	5	Describe the setting, locations, and relevant dates, including periods of recruitment, exposure, follow-up, and data collection	7
Participants	6	(*a*) Give the eligibility criteria, and the sources and methods of selection of participants	7
Variables	7	Clearly define all outcomes, exposures, predictors, potential confounders, and effect modifiers. Give diagnostic criteria, if applicable	7
Data sources/ measurement	8*	For each variable of interest, give sources of data and details of methods of assessment (measurement). Describe comparability of assessment methods if there is more than one group	7
Bias	9	Describe any efforts to address potential sources of bias	8
Study size	10	Explain how the study size was arrived at	
Quantitative variables	11	Explain how quantitative variables were handled in the analyses. If applicable, describe which groupings were chosen and why	8
Statistical methods	12	(*a*) Describe all statistical methods, including those used to control for confounding	8
		(*b*) Describe any methods used to examine subgroups and interactions	8
		(*c*) Explain how missing data were addressed	8
		(*d*) If applicable, describe analytical methods taking account of sampling strategy	8
		(*e*) Describe any sensitivity analyses	8
**Results**			
Participants	13*	(a) Report numbers of individuals at each stage of study—eg numbers potentially eligible, examined for eligibility, confirmed eligible, included in the study, completing follow-up, and analysed	9
		(b) Give reasons for non-participation at each stage	9
		(c) Consider use of a flow diagram	9
Descriptive data	14*	(a) Give characteristics of study participants (eg demographic, clinical, social) and information on exposures and potential confounders	9
		(b) Indicate number of participants with missing data for each variable of interest	9
Outcome data	15*	Report numbers of outcome events or summary measures	9
Main results	16	(*a*) Give unadjusted estimates and, if applicable, confounder-adjusted estimates and their precision (eg, 95% confidence interval). Make clear which confounders were adjusted for and why they were included	9

For peer review only

		(*b*) Report category boundaries when continuous variables were categorized	10
		(*c*) If relevant, consider translating estimates of relative risk into absolute risk for a meaningful time period	10
Other analyses	17	Report other analyses done—eg analyses of subgroups and interactions, and sensitivity analyses	10
**Discussion**			
Key results	18	Summarise key results with reference to study objectives	14
Limitations	19	Discuss limitations of the study, taking into account sources of potential bias or imprecision. Discuss both direction and magnitude of any potential bias	17
Interpretation	20	Give a cautious overall interpretation of results considering objectives, limitations, multiplicity of analyses, results from similar studies, and other relevant evidence	18
Generalisability	21	Discuss the generalisability (external validity) of the study results	18
**Other information**			
Funding	22	Give the source of funding and the role of the funders for the present study and, if applicable, for the original study on which the present article is based	18

*Give information separately for exposed and unexposed groups.

Note: An Explanation and Elaboration article discusses each checklist item and gives methodological background and published examples of transparent reporting. The STROBE checklist is best used in conjunction with this article (freely available on the Web sites of PLoS Medicine at http://www.plosmedicine.org/, Annals of Internal Medicine at http://www.annals.org/, and Epidemiology at http://www.epidem.com/). Information on the STROBE Initiative is available at www.strobe-statement.org.

For peer review only
